# Techno-economic and environmental feasibility of large-scale hybrid renewable energy system for coastal megaprojects: a case study of Ras El-Hekma, Egypt

**DOI:** 10.1038/s41598-026-58436-8

**Published:** 2026-06-22

**Authors:** Ahmed Sobhy, Mona Mohamed, Ahmed Kalas, Medhat H. Elfar

**Affiliations:** Department of Electrical Engineering, Faculty of Engineering, Port Said University, Port Said, 42526 Egypt

**Keywords:** Techno-economic analysis, Hybrid renewable energy systems, HOMER pro, Cost of energy, CO_2_ emissions, Climate sciences, Energy science and technology, Engineering, Environmental sciences, Environmental social sciences

## Abstract

This research provides a techno-economic and environmental evaluation of a large-scale hybrid renewable energy system (HRES) to support sustainable coastal development in Egypt, considering Ras El-Hekma as a representative case study. Six hybrid system configurations, including both grid-connected and off-grid modes, are modeled and evaluated using HOMER Pro. A large-scale load profile is developed with a peak demand of 28.8 MW and an annual electricity consumption of approximately 100.46 GWh over a 20-year project lifetime. The simulation results indicate that the optimal configuration is a 15 MW grid-connected wind energy system, achieving a Cost of Energy (COE) of $0.03409/kWh and a Net Present Cost (NPC) of $37.46 million with an initial investment cost of $24.8 million. The proposed system also achieves a renewable energy fraction of 68.6% and reduces CO₂ emissions by 60.45% compared with the base case grid-only scenario. Sensitivity analysis reveals that wind speed, project lifetime, inflation rate, and discount rate are the most influential factors affecting system feasibility and economic viability. The results demonstrate the strong potential of large-scale HRESs for sustainable energy development across Egypt’s coastal megaprojects.

## Introduction

The accelerating consumption of fossil fuels has resulted in severe environmental and public health challenges^1^. Transitioning to cleaner renewable energy sources is therefore essential to mitigate these impacts and support long-term global sustainability^2,3^. In addition to their technical and economic advantages, renewable energy technologies such as photovoltaic and wind energy systems provide significant positive social and environmental impacts through reducing greenhouse gas emissions, improving energy security, supporting sustainable development, and enhancing socio-economic growth^4,5^. By the end of 2025, the global renewable energy market has experienced remarkable growth, with the total installed renewable energy capacity exceeding 4,448 GW, including approximately 2,200 GW of solar photovoltaic systems and nearly 1,320 GW of wind energy installations. In parallel, the cumulative installed capacity of battery energy storage systems reached approximately 150 GW/363 GWh, highlighting the accelerating global transition toward sustainable and low-carbon energy systems^6^.

In many countries, the adoption of renewable energy technologies is becoming a global trend due to increasing environmental concerns and the growing demand for sustainable energy systems^7^. Egypt is actively aligning with this global energy transition. The country has extensive land availability together with strong solar irradiation and favorable wind conditions, which make it highly suitable for large-scale renewable energy development^8^. Wind speeds of 8–10 m/s in the Sinai Peninsula and the Gulf of Suez underscore the significant national potential for wind power generation^9^. These conditions enable the deployment of cost-effective wind power plants, establishing Egypt as a regional leader in wind energy. Solar conditions are equally favorable, with annual sunshine exceeding 3,000 h and total solar irradiance ranging from 2,000 to 3,200 kWh/m²^10,11^. These resources support the adoption of both photovoltaic (PV) systems and concentrated solar power (CSP). Under Egypt Vision 2030 and its extended energy roadmap, renewable sources are expected to supply 42% of national electricity generation by 2035, with wind and solar contributing 14% and 25%, respectively^12^.

As part of its ambitious Egypt Vision 2030 for economic transformation, the country is steadily advancing along a path of development and modernization. Among the new national mega-projects, the Ras El Hekma^13^ development on the Northwestern Mediterranean coast stands out as particularly strategic. With projected investments exceeding $35 billion, Ras El Hekma aims to become a global hub for urban development, tourism, and economic activity, with the first phase scheduled for completion by the end of 2028^14^. This rapid development is expected to drive a substantial increase in electricity demand, highlighting the importance of efficient energy systems. Accordingly, a comprehensive analysis is essential for identifying the most suitable energy configuration for such projects.

Determining the optimal configuration is a complex task, as the system may depend on solar energy, wind energy, energy storage systems, grid-connected configurations, or hybrid renewable energy systems^15^. Since numerous alternatives are available, each configuration must be assessed based on technical, economic, and environmental criteria to identify the most feasible and sustainable option for the study area. This process may involve minimizing the overall energy cost, maximizing environmental benefits, or achieving a balance between both objectives. Due to the complexity of these evaluations, specialized simulation and optimization tools are widely employed^16^.

Recent studies have increasingly focused on the techno-economic and environmental assessment of HRES. Hydrogen-supported energy systems have demonstrated promising capabilities for improving grid stability and covering electricity shortages in sub-grid applications^17^. In addition, the performance prediction and simulation of PV and wind systems under different climatic conditions is investigated to improve system reliability and operational efficiency^18^. The integration of pumped hydropower storage with renewable energy systems has also received considerable attention due to its ability to enhance energy management, reduce curtailment, and improve grid sustainability^19,20^. Moreover, techno-economic studies on large-scale grid-connected HRES confirmed the significant potential of renewable energy systems in reducing energy costs and greenhouse gas emissions while improving long-term energy security^21,22^.

On the other hand, several optimization tools are widely used to assess the techno-economic feasibility and environmental impact of hybrid renewable energy systems (HRES). These tools can be categorized into two main types: built-in optimization software, such as HOMER (Hybrid Optimization of Multiple Energy Resources)^23^, iHOGA (Improved Hybrid Optimization by Genetic Algorithm)^24^, SAM (System Advisor Model)^25^, and RETScreen (Renewable-energy and Energy-efficiency Technology Screening) Expert^26^, and custom optimization platforms such as MATLAB, where user-defined economic and environmental objective functions can be directly implemented^27^. Both categories are commonly employed to optimize the design, performance, and cost-effectiveness of HRES.

Among these, HOMER Pro stands out as a particularly prevalent platform in academic research^28^. It also boasts widespread practical implementation by over 250,000 system designers and developers across more than 190 countries^29^. This is because HOMER Pro incorporates three powerful analysis tools^30^. It simulates all feasible equipment combinations and models the operation of a hybrid microgrid over a full year^31^. Its proprietary HOMER Optimize algorithm identifies the least-cost solutions^32^. Several studies have employed HOMER Pro to address a wide range of objectives, including minimizing system cost, reducing carbon emissions, decreasing unmet load, maximizing overall system reliability, and enhancing energy efficiency. A summary of the reviewed literature is presented in Table 1.

A comprehensive techno-economic and environmental analysis of a fully HRES for seaport infrastructure in Somalia was carried out using HOMER Pro^33^. Their work integrated Pumped Hydro Energy Storage (PHES) as an alternative to conventional battery storage, which often suffers from limited lifetime, higher cost, and environmental concerns. The results indicated that the PV/WT/PHES configuration achieved strong economic performance with a net present cost (NPC) of $619.72 k, a Cost of Energy (COE) of $0.03845/kWh, and a short payback period of 0.31 years. Environmentally, the system produced approximately 1,029 tons of emissions annually. The feasibility of optimizing a nano-grid street lighting system, comprising PV, wind energy, and battery storage, using HOMER Pro was investigated^34^. This study aimed to improve energy efficiency while simultaneously lowering energy expenditures and greenhouse gas (GHG) emissions. Consequently, the selected optimal configuration yielded a discounted payback period (DPP) of 13 years, alongside an internal rate of return (IRR) of 5.5% and a net present value (NPV) of 45,820 USD.

For the industrial sector in Pakistan, an optimal hybrid energy system aimed at delivering an affordable and eco-friendly solution to foster industrial development was designed^35^. The study examined three distinct configurations, which included the existing energy setup (Case I), an on-grid biogas system (Case II), and an on-grid PV system integrated with batteries (Case III). The results indicated that Case III outperforms the other configurations, achieving a reduced Net NPC of $19.2 million, a COE of $0.034/kWh, and an Operating Cost (OC) of $573,371 per year. Furthermore, the on-grid PV system with batteries (Case III) offered significant environmental benefits, reducing CO₂ emissions by 63.82% and NOₓ emissions by 62.22%. A techno-economic analysis was conducted in Douala^36^, the capital of Cameroon, to identify the optimal hybrid energy system configuration for different consumer categories: low, medium, and high. The main objective was to minimize costs and eliminate unmet load, addressing the ongoing significant energy crisis in Cameroon. The study further examined electricity consumption and billing characteristics in Douala’s grid-connected energy systems. The best configuration (PV/BESS/DG/G) showed considerable cost reductions per kilowatt-hour, reaching 20.7%, 31.19%, and 63.49% for low, medium, and high consumption categories, respectively.

A techno-economic and environmental analysis to determine the optimal hybrid system for supplying electricity to long-term evolution (LTE) networks in remote areas was conducted^29^. To perform the assessment, the actual electricity consumption of LTE loads was analyzed alongside the specific geographical location and its corresponding climatic zone. The study compared four hybrid configurations: PV/BESS, PV/DG/BESS, WT/BESS, and PV/BESS/G. The PV/BESS configuration emerged as the optimal choice, yielding superior cost savings and the most substantial positive environmental impact. A techno-economic analysis in a region located in the northern part of Kandahar City, Afghanistan, was performed to identify a cost-effective and high-performing microgrid^37^. Six hybrid configurations, including PV/WT/BESS/DG, were compared. The results showed that the PV/DG/BESS configuration achieved the lowest NPC of $10,108 and a COE of $0.190/kWh.

A techno-economic-environmental analysis to determine the optimal hybrid system for the Baluchistan seashore was conducted^38^. The study designed a grid-connected system integrating wind turbines, solar PV, and converters, which reduces the amount of electricity purchased from the grid. The main objectives were to minimize operating costs, reduce NPC, and lower gas emissions. Four configurations were analyzed: PV/WT/DG/G, PV/G, WT/G, and G. With an annual operating cost reduction of $66,405 and a 64% cut in gas emissions, the PV/WT/DG/G setup was identified as the best-performing configuration. An optimized off-grid microgrid for a remote area was developed in Egypt, integrating solar PV, wind turbines, batteries, and a standby biomass generator to meet year-round power demand^39^. The system reduced reliance on conventional energy and lowered greenhouse gas emissions to 28,516 tons per year. It also achieved a competitive total NPC (USD 3,483,519) and a COE (USD 0.118/kWh), demonstrating both environmental and economic benefits.

Eight hybrid renewable energy system models, including PV, WT, BESS, BIOMG, and converters, were studied in the New Administrative Capital, New Cairo, Egypt^40^. The systems were designed, simulated, and optimized to meet the load of an international school. Among these, the PV/WT/BESS/BIOMG system with a converter was the most cost-effective. A hybrid system integrating wind turbines and micro-hydro power was designed and simulated to provide clean energy for purifying water in isolated settlements^41^. Its core purpose was to lower carbon emissions, as numerous remote locations continue to depend on diesel and other conventional fuels that pose environmental risks.

Despite this substantial body of work, limited research has examined hybrid energy systems for large coastal megaprojects such as Ras El Hekma. Furthermore, most existing research focuses on a single system configuration rather than comparing the relative performance of on-grid and off-grid hybrid systems. Limited attention has been given to applying these methodologies to major coastal development zones near national grid infrastructure, where both off-grid and grid-connected solutions are technically feasible. Additionally, previous studies rarely provided a systematic comparison between grid-connected and standalone systems based on site-specific resource availability. Therefore, for the Ras El-Hekma region, six wind-centric hybrid energy scenarios, encompassing both standalone and grid-tied setups, are rigorously assessed in this work to fill the identified research voids. The scenarios are evaluated in terms of renewable energy share, NPC, COE, supply adequacy, and emissions.

This study offers the following main contributions:

- A long-term meteorological characterization of Ras El-Hekma is conducted using continuous climatic records spanning 21 years.
- A comprehensive techno-economic-environmental comparison of on-grid and off-grid hybrid configurations is conducted for coastal megaprojects in Ras El-Hekma.
- Six system architectures integrating wind, solar, battery storage, diesel backup, and grid interaction are analyzed to determine the optimal configuration under local conditions, based on the most cost-effective option and the greatest environmental benefits.
- Sensitivity analysis is presented, examining the combined effects of inflation and interest rates on system viability.

A detailed description of the study location and its climatic conditions, specifically wind patterns, solar irradiance, and ambient temperature, is provided in Sect. “Study site description”. Section “Methodology” then delineates the methodological framework employed in this investigation. Section “Results and discussions” presents and discusses the simulation results, covering technical performance, economic viability, environmental impact, and sensitivity analysis. Section “Conclusion” concludes the study by summarizing the key findings.

**Table 1:** Summary of recent studies comparing optimization tools.

Ref.	Study Area	Mode	Objective Function	Optimal Configuration	Key Findings
^29^	October City, Egypt	Off Grid	Cost & Emissions	PV/BESS	A standalone PV/BESS system achieved 100% renewable energy fraction while meeting the mobile communication load with a low total system cost.
^33^	Somalia	Off Grid	Cost & Emissions	PV/WT/PHES	Achieve 100% renewable energy supply with a very low COE of $0.03845/kWh and significant CO₂ reduction.
^34^	Thailand	Off Grid	Economic Viability	PV/WT/BESS	Acceptable financial indicators (DPP 13 years, IRR 5.5%).
^35^	Pakistan	On Grid	Cost, Emissions, Reliability	PV/G/BESS	Minimize energy cost ($0.034/kWh) while reducing CO₂ and NOₓ emissions by more than 60%.
^36^	Douala, Cameroon	On Grid	Cost & Reliability	PV/DG/G	Provide a reliable and cost-effective energy supply for different consumer sizes with a COE ranging from $0.062 to $0.088/kWh.
^37^	Kandahar, Afghanistan	Off Grid	Cost & Reliability	PV/DG/BESS	Provide reliable off-grid electricity with a COE of $0.190/kWh and moderate investment cost.
^38^	Pakistan	On Grid	Cost & Emissions	PV/WT/G	Reduce emissions (64%) while lowering operational costs.
^39^	Egypt	Off Grid	Cost & Emissions	PV/WT/BESS/BIOMG	Supply high energy demand while reducing greenhouse gas emissions and maintaining acceptable economic performance.
^40^	New Cairo, Egypt	Off Grid	Cost & Emissions	PV/WT/BESS/BIOMG	Achieve a reliable power supply for a large institutional load, although with relatively high capital cost and COE.
^41^	Newfoundland and Labrador, Canada	Off Grid	Cost & Emissions	PV/WT/BESS/Hydro	Reduce CO₂ emissions while meeting the required load demand.

## Study site description

**Fig. 1:**
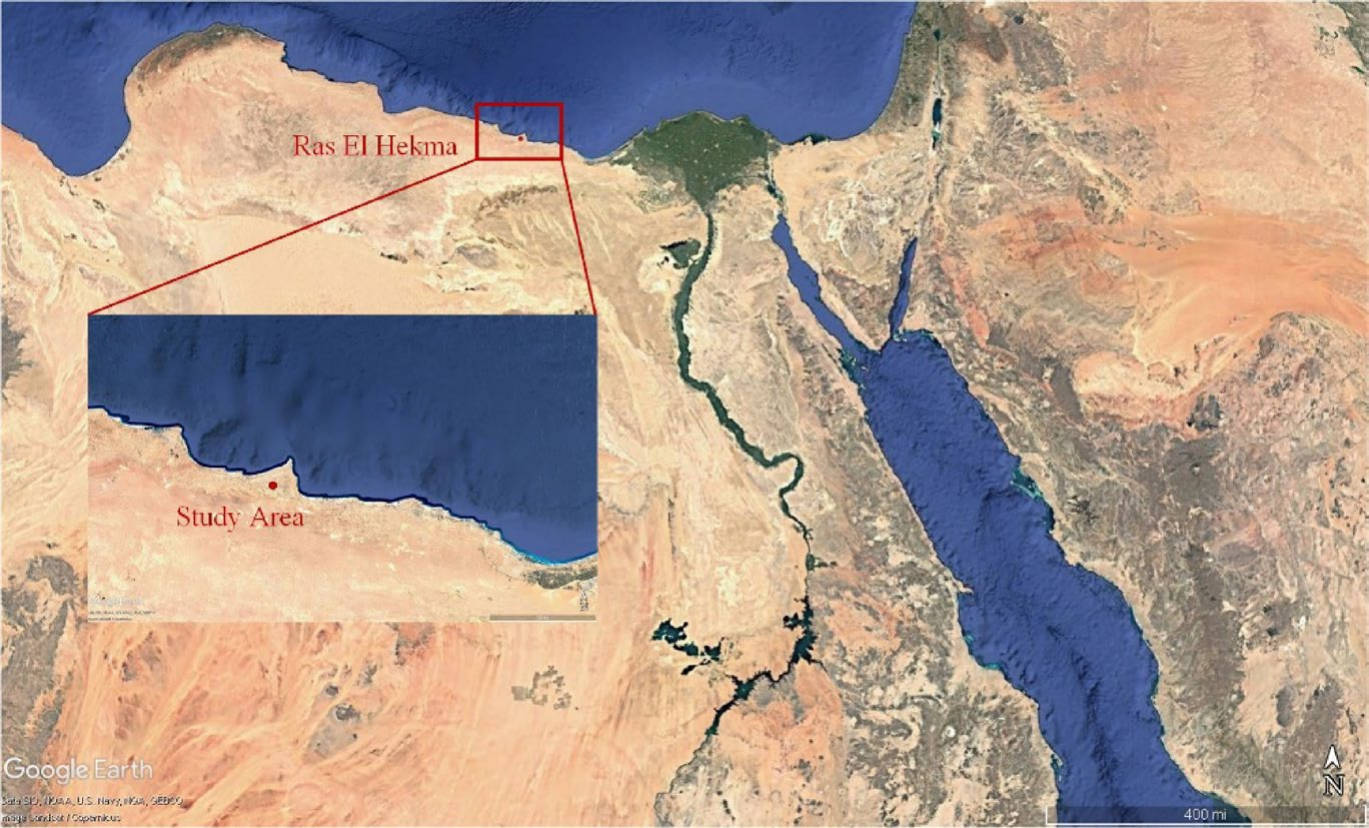
Geographic location of the study area at Ras El-Hekma, Egypt. The figure was generated using Google Earth Pro version 7.3.7 (https://www.google.com/earth/about/versions/#earth-pro). Satellite imagery and map data credits: Google, Landsat/Copernicus, SIO, NOAA, U.S. Navy, NGA, and GEBCO.

Ras Al-Hekma lies along Egypt’s northern Mediterranean coast. It is located between latitudes 31°16′5.79″N and 31°5′41.46″N, and longitudes 27°55′45.98″E and 27°41′5.64″E, as illustrated in Fig. 1. The specific coordinates of the study are 31° 8′ 24″ N and 27° 46′ 48″ E.

### Wind resource data

**Fig. 2:**
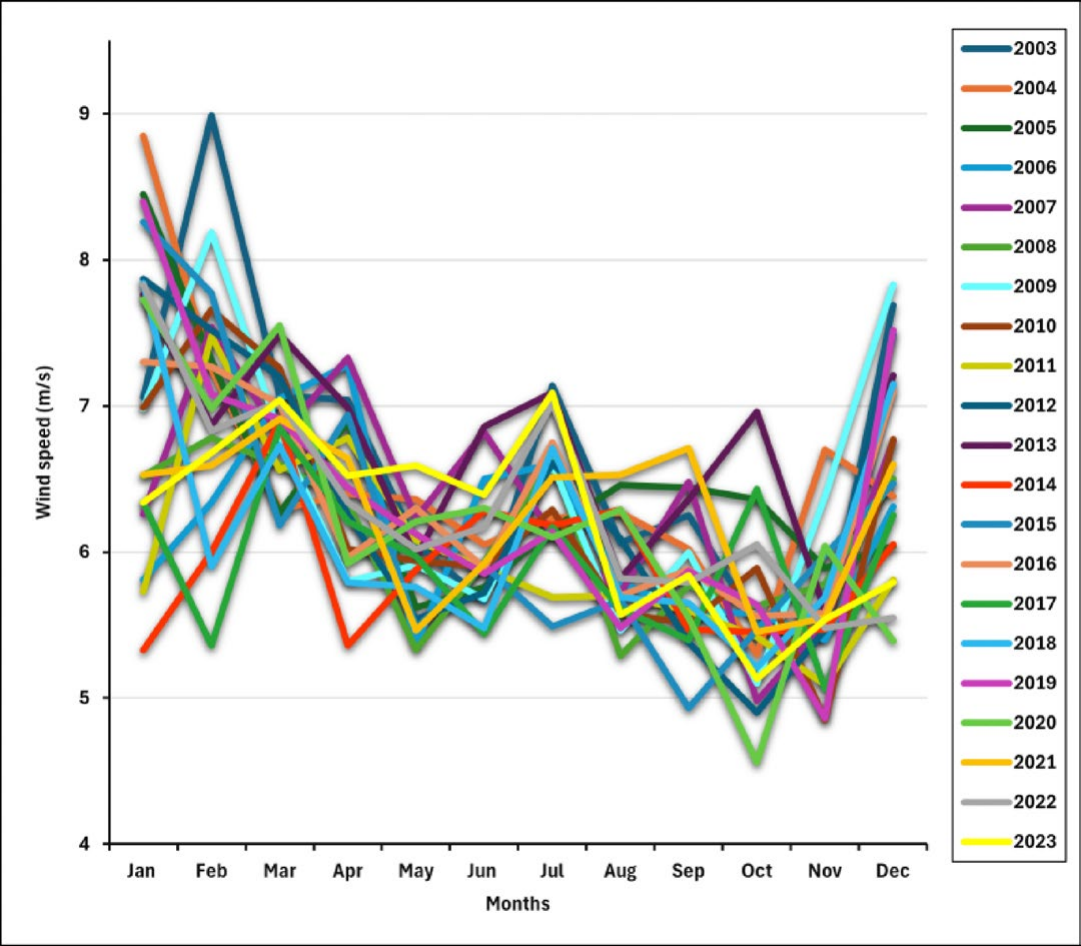
Monthly average wind speed at 50 m 2003/01/01 to 2023/12/31 [Lat: 31.14, Lon: 27.78].

**Fig. 3:**
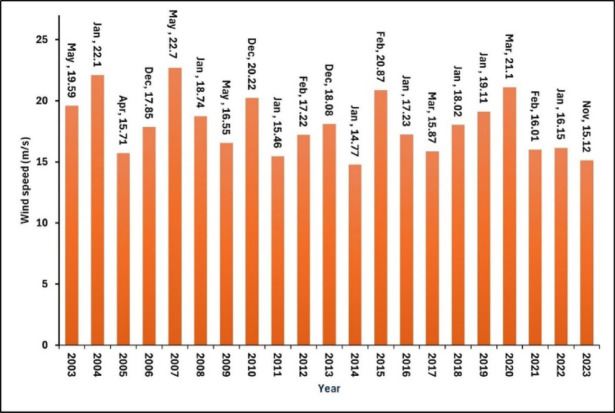
Monthly maximum wind speed at 50 m 2003/01/01 to 2023/12/31 [Lat: 31.14, Lon: 27.78].

A long-term study is conducted for the period from January 1, 2003, to December 31, 2023, using data from the NASA POWER Project, which provides continuous climatic records over 21 years. The analysis primarily focuses on the variation of wind speed at a reference height of 50 m above sea level. The results indicate that the wind resource in Ras El Hekma is stable, as shown in Fig. 2, with an average wind speed of 6.19 m/s over the study period. The highest recorded wind speed was 22.7 m/s in May 2007, as illustrated in Fig. 3.

**Fig. 4:**
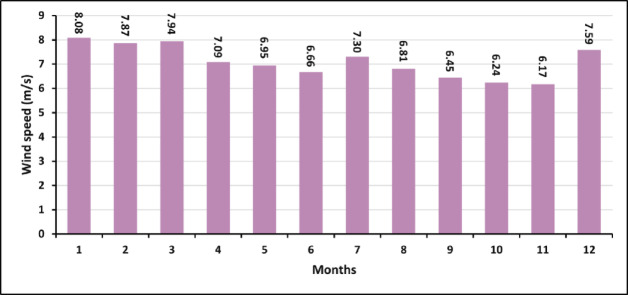
Monthly average wind speed at 100 m above the surface of the sea.

**Fig. 5:**
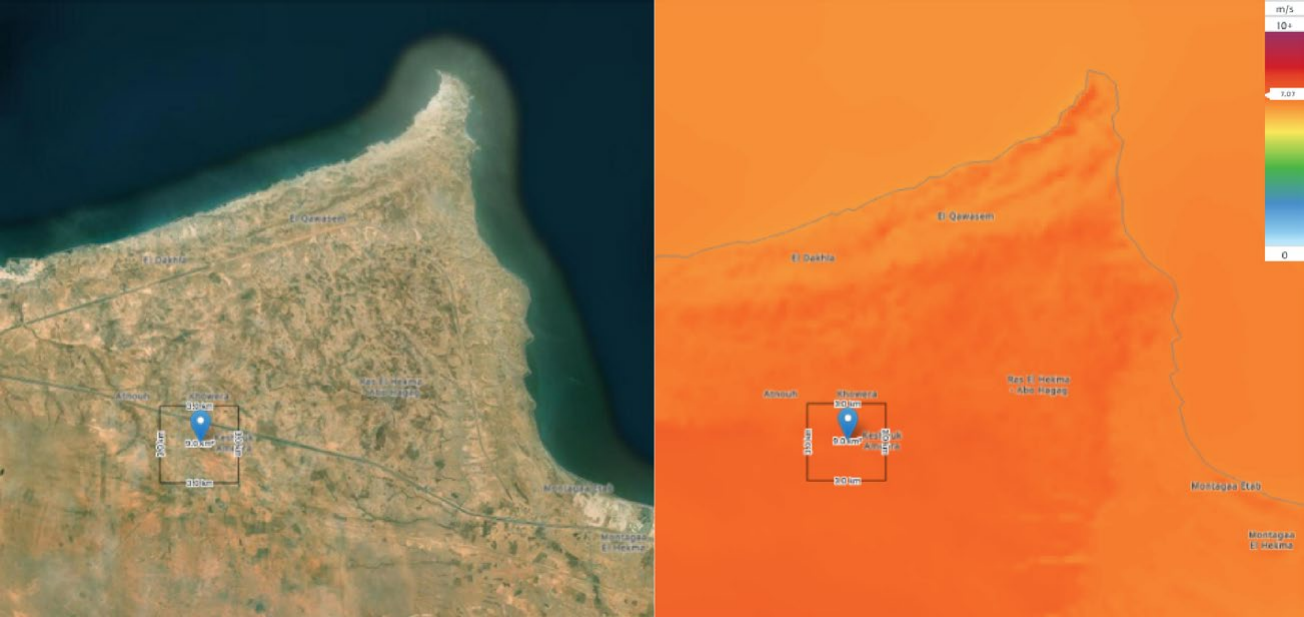
Mean wind speed at 100 m – Global Wind Atlas.

In addition to NASA data, wind resource information from the Egyptian Wind Atlas is used to evaluate the wind potential at Ras El-Hekma at a height of 100 m. The monthly average wind speed at this elevation is shown in Fig. 4, reaching 7.09 m/s, with a corresponding power density of 315 W/m². These values indicate that Ras El-Hekma has excellent wind resources at this elevation, as shown in Fig. 5.

### Solar resources data

**Fig. 6:**
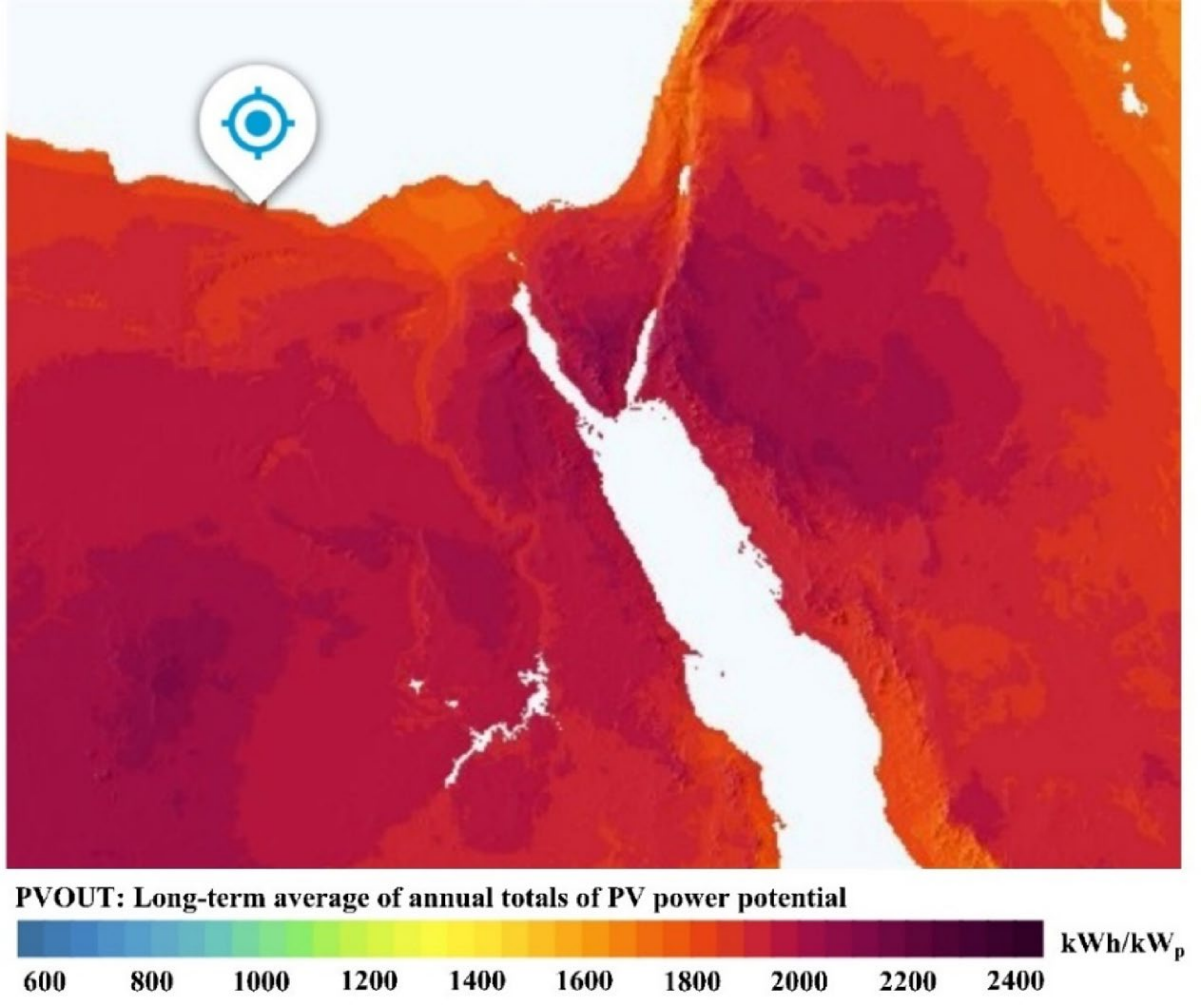
Egypt’s PVOUT map, showing the region with the high-power potential values.

**Fig. 7:**
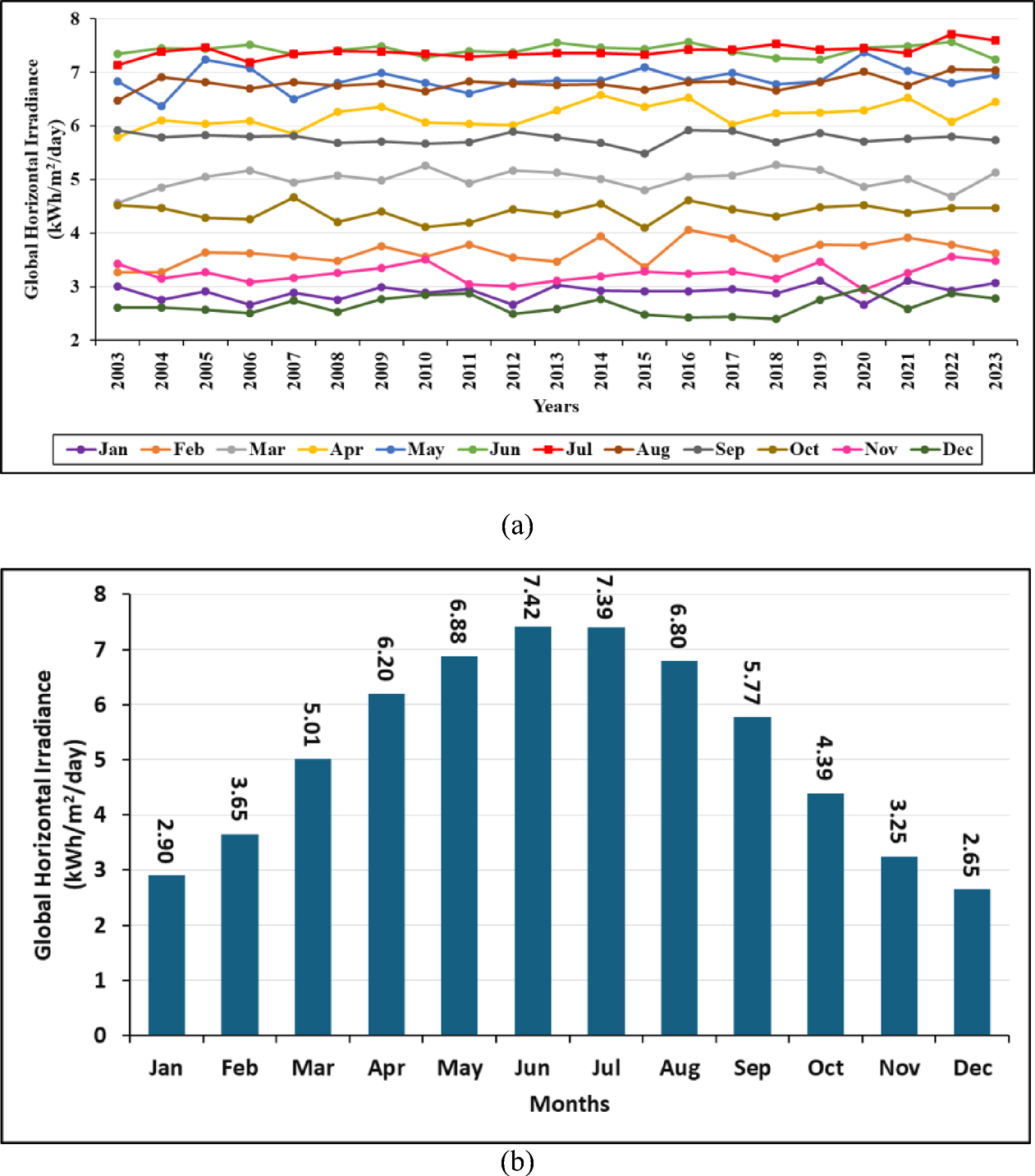
Global horizontal irradiance (kWh/m^2^/day) data: **(a)** Monthly diffuse horizontal irradiance (2003–2023); **(b)** Monthly average.

**Fig. 8:**
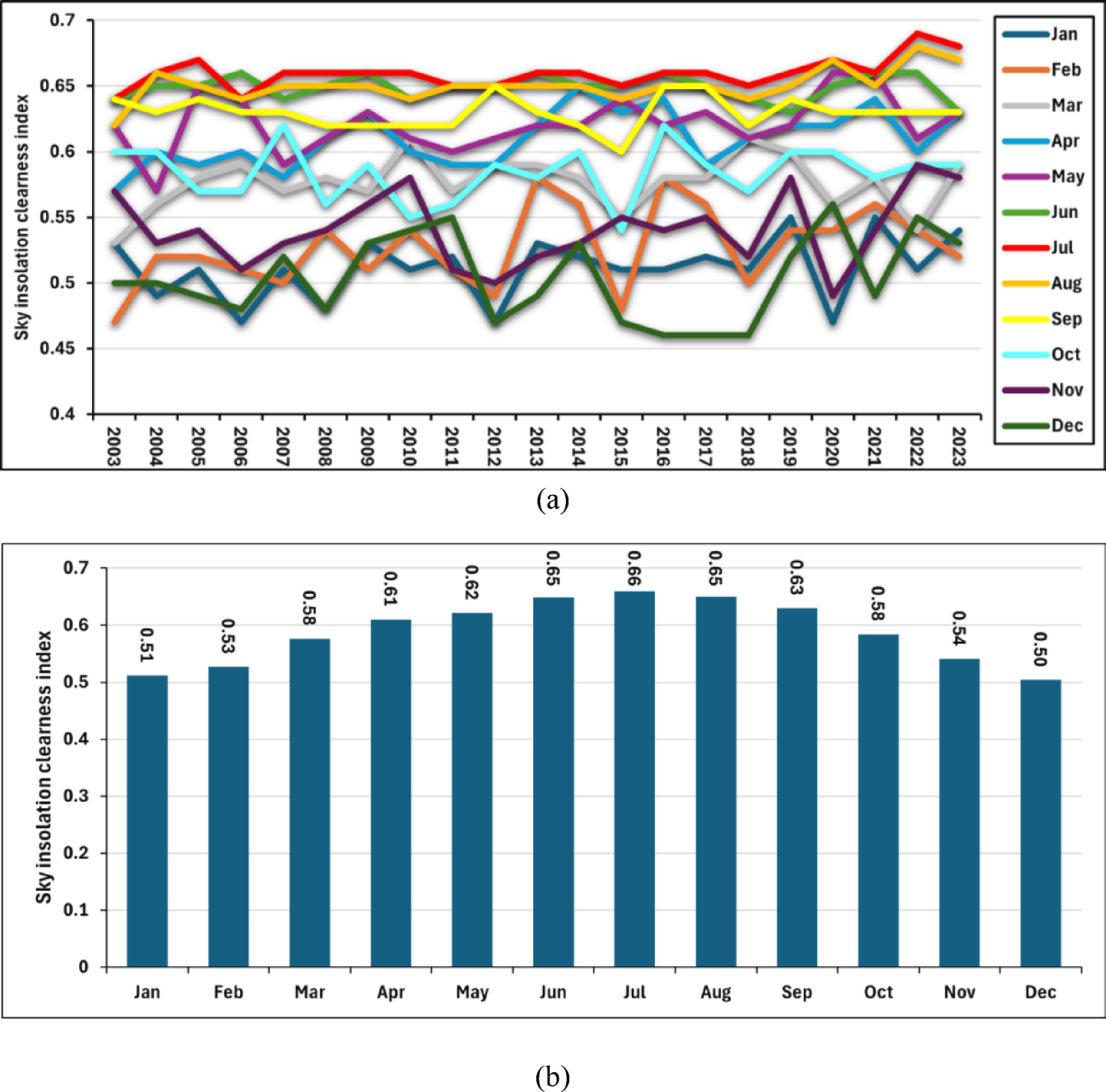
Sky insolation clearness index data: **(a)** Monthly sky insolation clearness index (2003–2023) **(b)** Monthly average.

A detailed solar resource assessment is conducted to evaluate the suitability of photovoltaic (PV) systems in the Ras El-Hekma region. According to the Solar Atlas, the study area lies within a high-yield solar zone with an average PVOUT of 1,841.9 kWh/kWp, indicating strong solar energy potential, as shown in Fig. 6. Table 2 summarizes the monthly and annual solar irradiation characteristics, with a yearly global horizontal irradiance (GHI) of 2,074.7 kWh/m² and an optimum PV tilt angle of 30$\:^\circ\:$. In addition, a long-term analysis using NASA POWER data from 2003 to 2023 is performed. Figures 7 and 8 present the GHI and clearness index variations, respectively, showing an average GHI of 5.193 kWh/m²/day and an average clearness index of 0.589. The results confirm the suitability of Ras El-Hekma for large-scale PV system deployment.

The adopted solar irradiation data sources and assessment approach are consistent with methodologies commonly employed in previous studies related to solar irradiance evaluation and photovoltaic system analysis under different climatic conditions^42,43^.

**Table 2:** Measured solar irradiation at the study site.

Parameter	Value	Unit
Diffuse irradiance (DFI)	724.2	kWh/m^2^
Global horizontal irradiance (GHI)	2,074.7	kWh/m^2^
Direct normal irradiance (DNI)	2,080.6	kWh/m^2^
Optimum tilt of PV module	30/180	degrees
Global tilted irradiation at the optimum angle	2,308.0	kWh/m^2^

### Temperature data

**Fig. 9:**
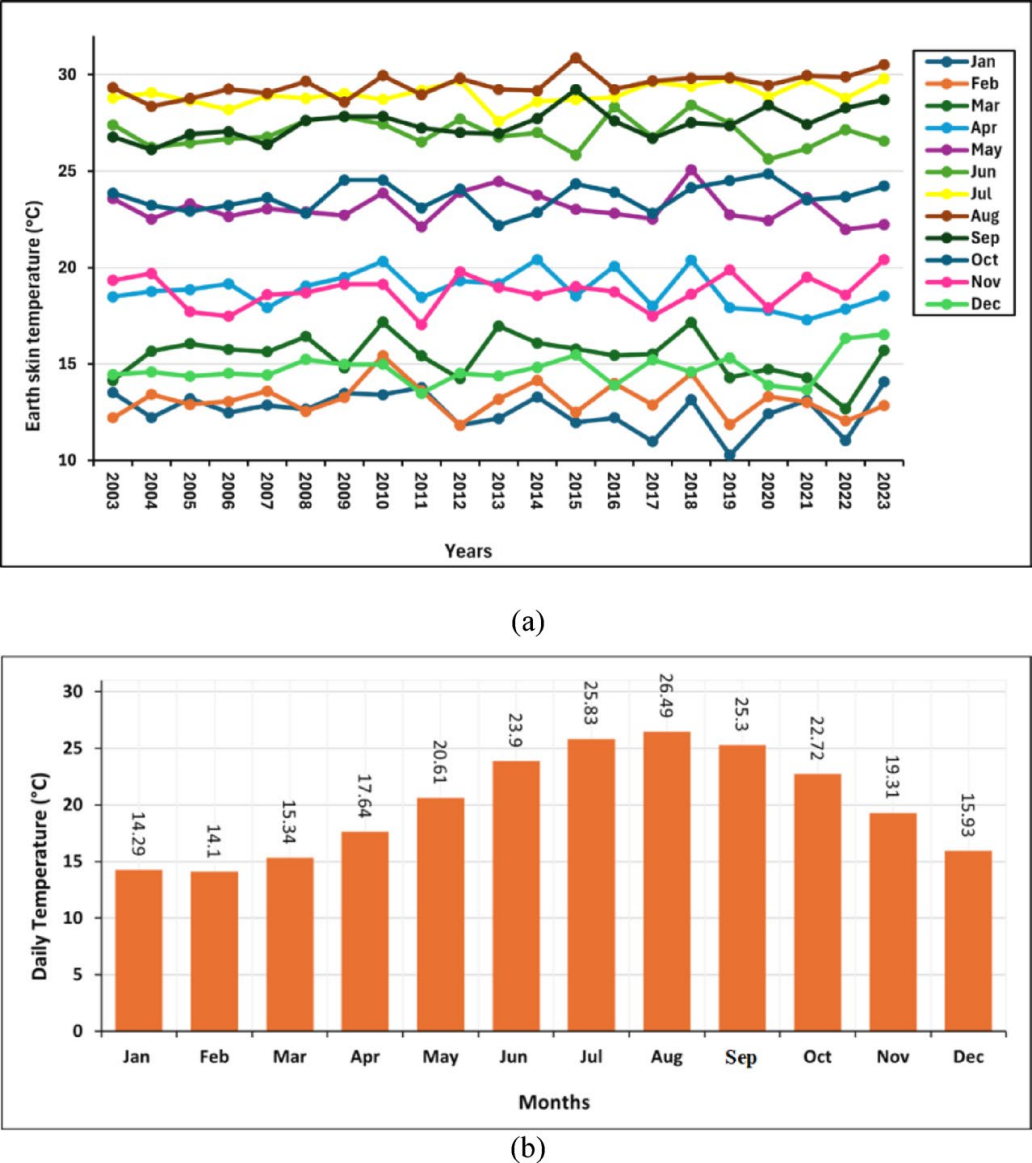
Temperature data: **(a)** monthly average temperature of the area (2003–2023). **(b)** monthly average temperature of the area.

As illustrated in Fig. 9(a), the region experiences a Mediterranean climate with hot, arid summers and mild, rainy winters, a pattern that remained consistent throughout the 2003–2023 study period using NASA POWER data. During this time, August emerged as the warmest month with average temperatures near 30.87 °C and peaks exceeding 40 °C, while January averaged a cooler 10.28 °C. The area’s mean annual temperature settled at approximately 20.12 °C, as depicted in Fig. 9(b). These ambient conditions carry significant implications for energy system performance: photovoltaic modules suffer efficiency losses from high heat due to silicon’s negative temperature coefficient^44,45^, while lithium-ion batteries undergo accelerated aging in high temperatures and diminished functionality in extreme cold^46^.

### Load profile

**Fig. 10:**
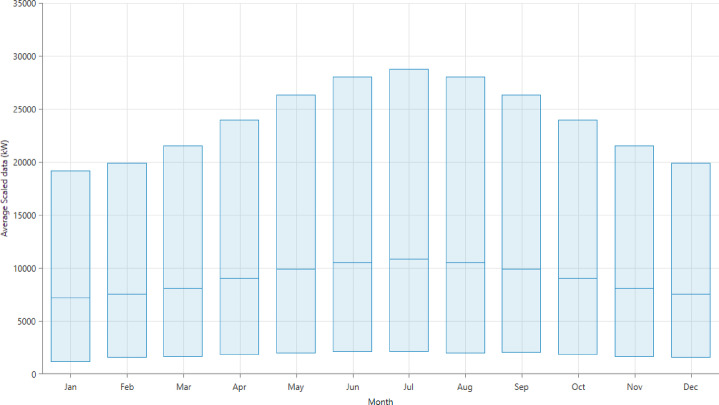
Scaled data monthly profile.

**Fig. 11:**
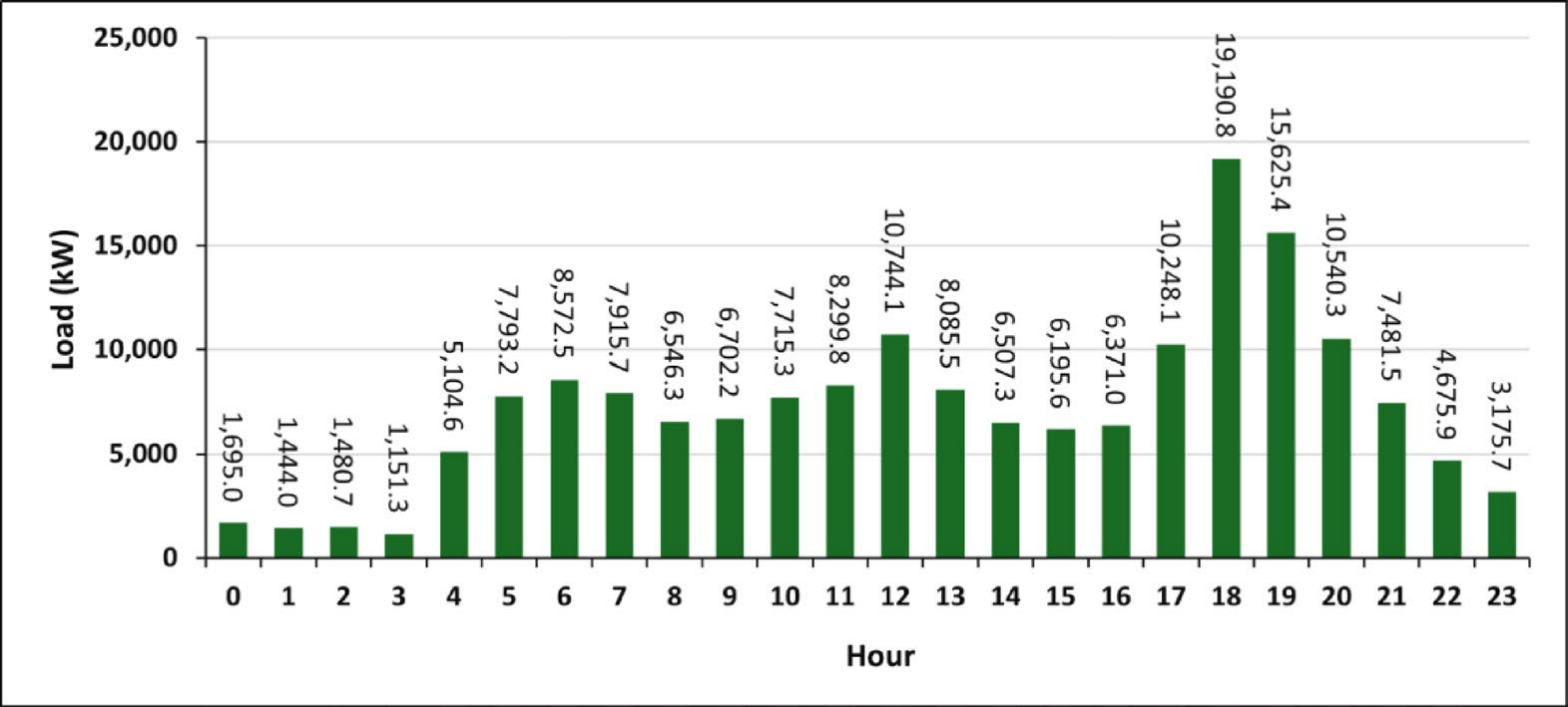
January daily profile.

The Ras El-Hekma development on the northwestern Mediterranean coast is a strategic project aimed at becoming a global hub for urban development, tourism, and economic activity. A representative daily electrical load profile is proposed based on the expected operational characteristics of the planned megaproject, considering the combined contribution of residential, tourism, commercial, desalination, and infrastructure-related activities together with the anticipated seasonal variation in occupancy and electricity demand throughout the year^47^. The development plan comprises several integrated resort complexes over an area of about 5,750 km², including luxury villas, hotels, commercial zones, desalination plants, recreational areas, and administrative infrastructure. The estimated load profile indicates a peak demand of 28.8 MW occurs in July at 18:00 h and an average load of 9.05 MW, with a base load of 1.444 MW sustained throughout the day. Small peaks of about 10.774 MW occur in the morning and at noon, while the highest energy consumption is observed in the evening, as shown in Fig. 10. Figure 11 represents the daily load profile in January.

## Methodology

**Fig. 12:**
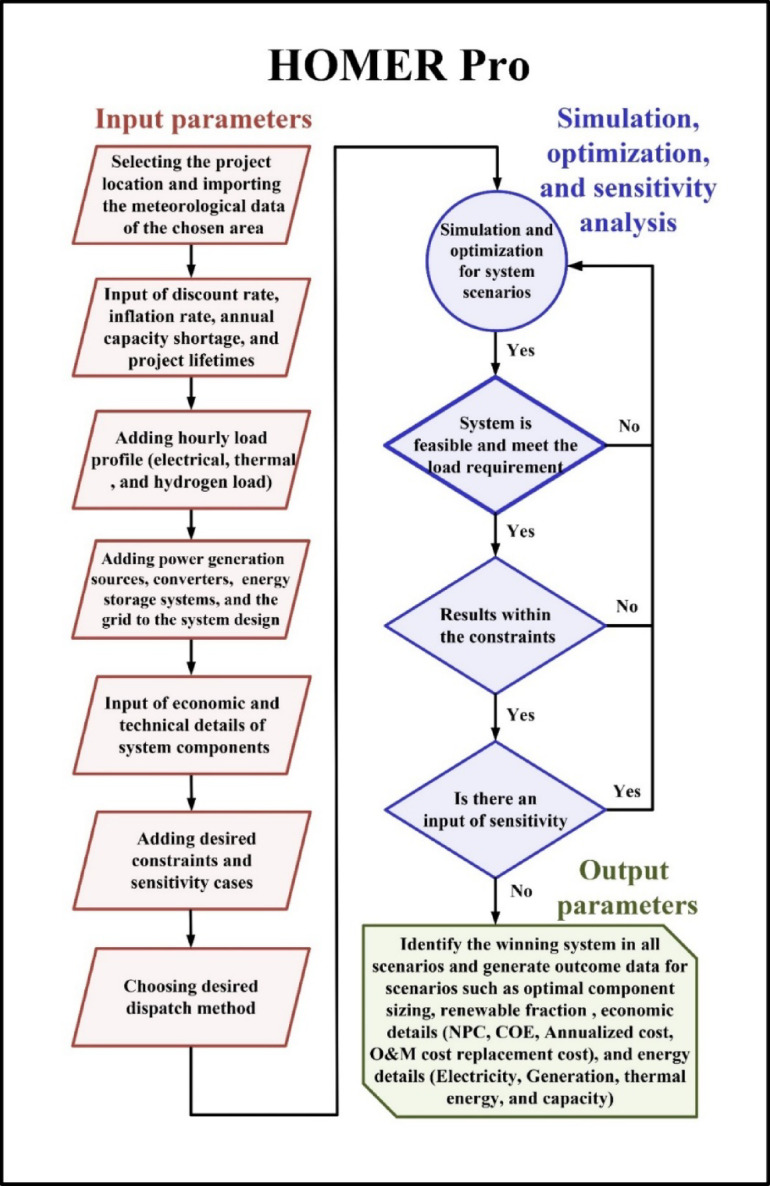
Flow diagram of optimization and simulation in Homer Pro.

To evaluate six potential energy configurations for Ras El-Hekma, the HOMER Pro platform is employed for simulation, capacity optimization, and sensitivity testing. Since HOMER Pro is a built-in optimization platform, the economic assessment and optimization procedures adopted in this study follow the standard mathematical and economic formulations internally implemented within the software environment. The modeled system integrated photovoltaic panels, wind turbines, a diesel generator, power conversion units, battery storage, and provisions for grid interconnection. Seeking the design with the minimum COE^48^, the analysis follows the structured workflow depicted in Fig. 12, a sequential process incorporating component specifications, resource availability, technical limitations, and financial inputs. Underlying these calculations are the economic formulations, which govern the software’s cost assessments as^49^:

1 $$\:NPC=\:\frac{{C}_{ann,total}}{CRF(i,\:{\:R}_{proj})}$$

2 $$\:CRF=\:\frac{i\:{(1+i)}^{N}}{{(1+i)}^{N}\:-\:1}$$

3 $$\:i=\:\frac{{i}^{{\prime\:}}-\:f}{1+f}$$

4 $$\:COE=\:\frac{{C}_{ann,tot}}{{E}_{Load\:Served}}$$

Where *NPC* denotes the total NPC of the system components ($), $$\:{C}_{ann,total}$$ total annualized cost ($/yr), $$\:{\:R}_{proj}$$ represents the project lifetime (years), $$\:i$$ is the real discount rate, *CRF* is the capital recovery factor, $$\:{CRF}_{\left(i,\:{R}_{proj}\right)}\:$$is the corresponding compensation factor, *N* is the number of years, $$\:{i}^{{\prime\:}}$$ is the nominal interest rate percent, $$\:f$$ is the expected inflation rate, $$\:{E}_{Load\:Served}$$ is the total annual electrical energy delivered by the system (kWh/yr), and $$\:COE$$ refers to the cost of energy ($/kWh).

**Fig. 13:**
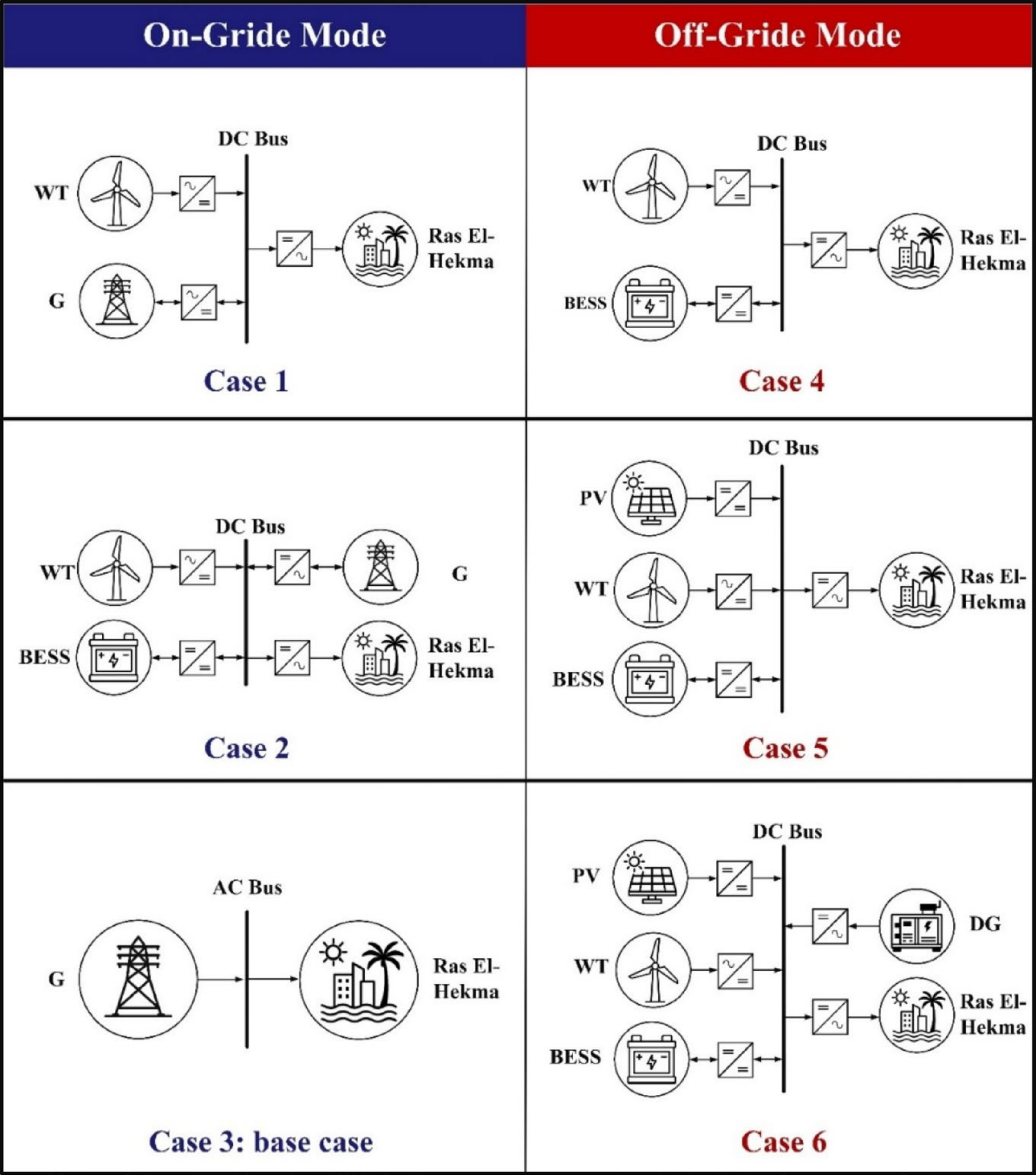
Schematic diagram of the cases.

Ras El-Hekma is not a remote area and has access to the national grid, in addition to being rich in both wind and solar resources. Therefore, multiple scenarios are valid, including both on-grid and off-grid modes. These scenarios are analyzed using HOMER Pro, as illustrated in Fig. 13; Table 3, to evaluate the techno-economic performance and identify the most cost-effective and reliable solution for sustainable power supply in Ras El Hekma.

**Table 3:** System combination.

Name	System	Mode	Description
Case 1	WT + G	On-Grid Mode	A wind turbine system connected to the national grid, serving as the primary source of power while relying on the grid for balancing supply and demand.
Case 2	WT + BESS + G		A wind turbine system connected to the grid with an integrated battery energy storage system (BESS) to enhance system reliability and handle fluctuations in generation.
Case 3	G (base case)		Complete reliance on the national grid with no contribution from renewable energy sources serves as the reference scenario for comparison.
Case 4	WT+ BESS	Off-Grid Mode	An off-grid configuration where wind turbines are combined with batteries to supply power independently, without any grid connection.
Case 5	WT + PV + BESS		A fully renewable hybrid system combining wind turbines, photovoltaic panels, and batteries, providing 100% renewable energy autonomy for off-grid operation.
Case 6	WT + PV + DG + BESS		A hybrid off-grid system integrating wind turbines, solar panels, batteries, and a diesel generator, with the generator acting as a backup to meet load demands when renewable sources and batteries are insufficient.

The analysis began with the on-grid scenarios. In the first case, the system combines wind turbines with a grid connection, as in case 1. In the second case, a battery storage system is integrated as a backup to enhance system reliability. The base case (third scenario) assumes complete dependence on the national grid to meet the total load demand.

Conversely, the off-grid scenarios explored different combinations of renewable energy sources (RESs), batteries, and diesel generators. The fourth case combines wind turbines with batteries to meet the load requirements. In the fifth scenario, photovoltaic panels are added to improve system reliability. The final case includes a diesel generator as a backup energy source when the load demand could not be met by either the renewable energy sources (RES) or the batteries, to assess its ability to satisfy the load requirements. These cases also highlight the practicality and cost-effectiveness of decentralized energy solutions compared with grid-connected systems.

### System modeling

In this section, a mathematical framework is developed for the proposed HRES, which integrates photovoltaic panels, wind turbines, a diesel generator, battery storage, and grid connectivity across six distinct configurations. The presented mathematical models are based on the component modeling framework implemented within the HOMER Pro software. Proper component selection suited to the study area’s characteristics is fundamental to determining the most viable system design. Accordingly, each element is modeled in detail, with particular attention given to its technical specifications, operational constraints, and associated economic parameters that influence overall system performance and feasibility.

#### Wind turbine model

To determine the wind speed at turbine hub height, the following expression is employed^50^:

5 $$\:{V}_{hub}={V}_{anem}\cdot\:{\left(\frac{{Z}_{hub}}{{Z}_{anem}}\right)}^{\alpha\:}$$

The output power of the wind turbine is determined as^51^:

6 $$\:{P}_{W}=\frac{1}{2}\:\rho\:{C}_{p}A\sum\:_{1}^{j}{f}_{V}\:{V}^{3}$$

where $\:{V}_{hub}$ denotes the wind speed at the turbine’s hub height (m/s), $\:{V}_{anem}$ is the wind speed measured at the anemometer height (m/s), $\:{Z}_{hub}$ represents the hub height of the wind turbine (m), $\:{Z}_{anem}$ is the height at which the anemometer is installed (m), $\:\alpha\:$ refers to the wind shear coefficient, $\:\rho\:$ is the air density (kg/m^3^), $\:{C}_{p}$ is the turbine’s power coefficient, $\:A$ is the swept area of the turbine blades (m^2^), $\:{f}_{V}$ is the wind velocity distribution, and $\:V$ is the wind velocity (m/s).

In practical operation, the generated wind turbine power is also governed by the turbine power curve, with cut-in, rated, and cut-off wind speeds. Accordingly, the wind turbine output power can be expressed as^52^:

7 $$P_{W} = \begin{cases} P_{rat} & V_{rat} \le V_{hub} \le V_{cut-off} \\ P_{rat} \left( \frac{V_{hub} - V_{cut-in}}{V_{rat} - V_{cut-in}} \right) & V_{cut-in} < V_{hub} < V_{rat} \\ 0 & V_{hub} \le V_{cut-in} \quad \mathrm{OR} \quad V_{hub} > V_{cut-off} \end{cases}$$

where $\:{P}_{rat}$ represents the rated power of the wind turbine at rated wind speed $\:{V}_{rat}$, $\:{V}_{cut-in\:}$ and $\:{V}_{cut-off\:}$ denote the cut-in and cut-off wind speeds, respectively.

**Fig. 14:**
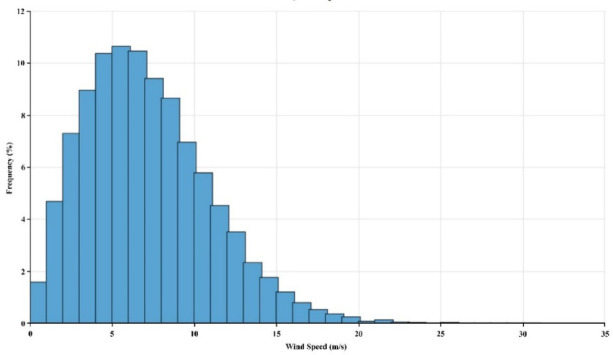
Wind speed frequency distribution at 100-meter hub height in the study area.

**Fig. 15:**
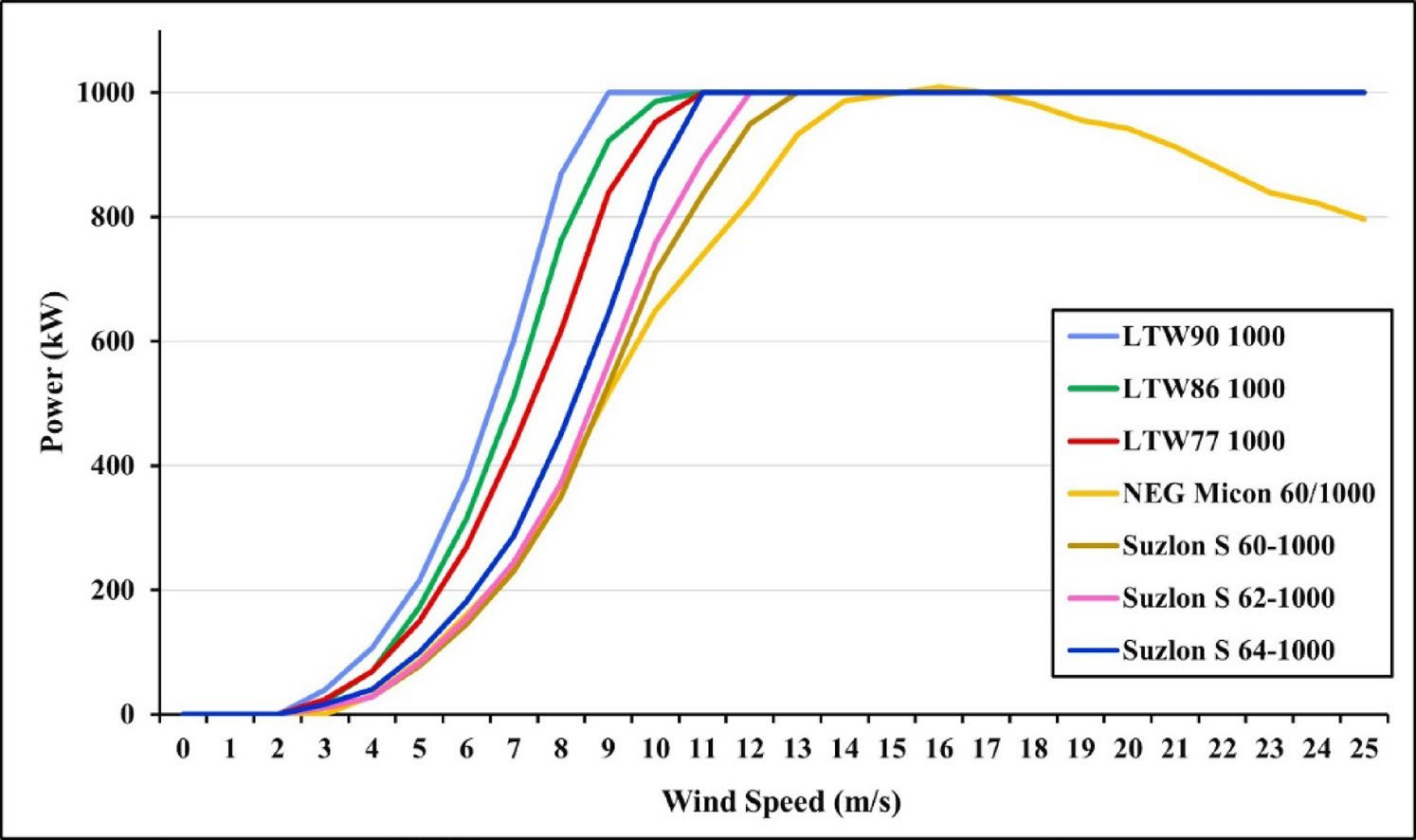
Comparison of actual power curves for different 1,000 kW wind turbines, based on data from manufacturer specifications.

As this project is classified as a large-scale wind energy development, the selection of an appropriate wind turbine is critical. For this analysis, a 1 MW-rated wind turbine is selected as the reference technology. Based on site-specific wind characteristics, a 100 m hub height is considered optimal for maximizing energy capture. Figure 14 presents the annual wind speed frequency distribution at this elevation, revealing that a velocity of approximately 6 m/s is the most prevalent, representing 10.63% of all observations throughout the year. Wind speeds above this value occur less frequently, with significantly lower probabilities. For instance, a wind speed of 13 m/s is observed only 3.49% of the time, indicating that such high speeds are relatively rare in this location. Consequently, a turbine with a low rated wind speed is required to ensure efficient energy capture under the prevailing wind conditions. Several commercially available wind turbine models are evaluated based on their power curves, as shown in Fig. 15. The Leitwind 90 1000 kW wind turbine meets these criteria and is therefore selected as the most suitable option for the site. In addition, its permanent magnet synchronous generator (PMSG) offers higher energy production at medium and low wind speeds compared to double-fed induction generators (DFIG)^53^. The power curve and technical specifications of the selected turbine are presented in Table 4.

**Table 4:** Leitwind 90 1000 kW features^54^.

Characteristics	Value	Unit
Model	Leitwind 90 1000 kW	-
Rated power	1,000	kW
Cut in speed	3	m/s
Rated speed	9	m/s
Cut out speed	25	m/s
Rotor diameter	90.3	m
Swept area	6,404	m²
Wake effect losses	5	%
Lifetime	20	yr
Generator voltage	690	V
Capital cost	1,650,000	$
Replacement cost	1,600,000	$
Operation and maintenance (O&M) cost	40,000	$/yr

#### PV module model

The output of the PV system under varying operating conditions can be determined as^33^:

8 $$\:{P}_{PV}={Y}_{PV}\times\:{f}_{PV}\left(\frac{{G}_{T}}{{G}_{T,\:\:STC}}\right)\left[1+{\alpha\:}_{P}\left({T}_{C}-{T}_{C,STC}\right)\right]$$

The PV cell temperature $\:{T}_{C}$ can be estimated using the Nominal Operating Cell Temperature ($\:\mathrm{N}\mathrm{O}\mathrm{C}\mathrm{T}$) approach as follows^55^:

9 $$\:{T}_{C}={T}_{amb}+\frac{{G}_{T}}{800}\left(\mathrm{N}\mathrm{O}\mathrm{C}\mathrm{T}-20\right)$$

where, $\:{Y}_{PV}$ signifies the nominal PV array capacity under standard test conditions, expressed in kilowatts, $\:{f}_{PV}$ represents the derating factor, which accounts for operational losses as a percentage, $\:{G}_{T}$ denotes the incident solar radiation on the array during each simulation interval ($\:W/{m}^{2}$), $\:{G}_{T,\:\:STC}$ denotes the reference irradiance at standard test conditions, $\:{\alpha\:}_{P}$ represents the temperature coefficient of power $\:\left(per\:^\circ C\right)$, $\:{T}_{C}$ denotes the cell temperature during the current time step $\:\left(^\circ\:\mathrm{C}\right)$, $\:{T}_{C,STC}$ is the cell temperature under standard test conditions $\:\left(^\circ\:\mathrm{C}\right)$, $\:{T}_{amb}$ is the ambient air temperature $\:\left(^\circ\:\mathrm{C}\right)$, and $\:\mathrm{N}\mathrm{O}\mathrm{C}\mathrm{T}$ represents the nominal operating cell temperature (°C)$\:.$ PV manufacturers typically report NOCT values for commercially available PV modules within a range of 45 °C to 48 °C, depending on the module design and operating characteristics^55^.

In this study, the 20 kW capacity Fronius Symo 20.0-3-M inverter, coupled with generic photovoltaic (PV) panels, is selected for the system design. The selected generic PV configuration is adopted to provide a representative and flexible modeling approach for large-scale photovoltaic system assessment under the climatic conditions of the study area, where the primary objective of the study is the techno-economic optimization of the hybrid energy system rather than the comparative evaluation of specific PV module technologies. In addition, the Fronius Symo 20.0-3-M inverter is selected due to its suitability for three-phase grid-connected photovoltaic applications, high operational reliability, and compatibility with the proposed HRES configuration considered in this study. The specifications of the PV panel are given in Table 5.

**Table 5:** Fronius Symo 20.0-3-M with Generic PV features^56^.

Characteristics	Value	Unit
Model	Fronius Symo 20.0-3-M	-
Rated capacity	20	kW
Power temperature coefficient	− 0.41	$$\:\mathrm{\%}/{^\circ C}$$
Normal operating cell temperature	45	$$\:{^\circ C}$$
Efficiency at STC	17.30	%
Derating factor	96	%
Lifetime	25	Yr
Capital cost	3,000	$
Replacement cost	3,000	$
Operation and maintenance (O&M) cost	10	$/yr

#### Converter model

In hybrid systems integrating both DC and AC elements, a bidirectional converter is essential for enabling two-way power transfer. Within the HOMER Pro environment, this component functions dually as an inverter and as a rectifier for the reverse process. For the present configuration, the SolarMax RX500 converter is selected, whose detailed technical specifications are provided in Table 6. The required converter capacity can be determined as^33^:

10 $$\:{P}_{con}=\frac{{P}_{peak}}{{\eta\:}_{con}}$$

where $\:{\eta\:}_{con}$ represents the efficiency of the converter, and $\:{P}_{peak}$ denotes the maximum demand level at the load point (kW).

**Table 6:** SolarMax RX500 converter features^47^.

Characteristics	Value	Unit
Model	SolarMax RX500	-
Maximum DC voltage	1,000	V
Maximum DC current	960	A
Nominal power	500	kW
Maximum AC current	920	A
Efficiency	98	%
Lifetimes	25	yr
Capital cost	590	$
Replacement cost	590	%
Operation and maintenance (O&M) cost	0.018	$/yr

#### Battery model

Among the various storage technologies, lead-acid and lithium-ion batteries are the most commonly employed^57^. Lead-acid variants have seen extensive use, especially in solar PV installations, owing to their dependable performance and low upfront costs^58^. This affordability has made them a dominant choice in regions like sub-Saharan Africa, where economic considerations often outweigh technological sophistication^59^. Lithium-ion batteries, by contrast, offer distinct advantages such as enhanced energy density, extended cycle life, reduced maintenance, and improved environmental profiles^60^. These characteristics position lithium-ion technology as a more sustainable and efficient option for long-duration storage applications. For this study, the Generic 4-hour 1 MW Li-Ion battery is chosen, as four-hour lithium-ion systems represent the most widely deployed storage solution in contemporary grids, effectively addressing peak summer demand while complementing solar generation. Their maturity, scalability, economic viability, and alignment with current regulatory frameworks render them the predominant choice in modern energy markets^61^. The technical and economic specifications of the selected Generic 4 h 1 MW Li-Ion battery storage system summarized in Table 7.

The battery state of charge ($\:{SOC}_{bat}$) during discharge and charge cycles can be determined as follows^62^:

11 $$\:{SOC}_{bat}(t+1)\:=\:{SOC}_{bat}\left(t\right)\:\times\:\:(1\:-\:\sigma\:)\:-\:\:\left(\frac{{E}_{l}\left(t\right)}{{\eta\:}_{cnv}}\:-\:{E}_{g}\left(t\right)\right)\times\:\:{\eta\:}_{BD}$$

12 $$\:{SOC}_{bat}(t+1)\:=\:{SOC}_{bat}\left(t\right)\:\times\:\:(1\:-\:\sigma\:)\:-\left({E}_{g}\left(t\right)-\:\frac{{E}_{l}\left(t\right)}{{\eta\:}_{cnv}}\:\right)\:\times\:\:{\eta\:}_{BC}$$

The number of battery units required is determined as^63^:

13 $$\:{N}_{bat}\:=\frac{{P}_{avg}\:AH}{{C}_{bat}\:{\eta\:}_{bat}\:DOD}$$

where $\:{E}_{l}\left(t\right)$ is the energy demand (kWh), while $\:{E}_{g}\left(t\right)\:$is generated power (kWh). The terms $\:{\eta\:}_{BD}$ and $\:{\eta\:}_{BC}$ denote the efficiencies associated with battery discharging and charging, respectively, while $\:{\upsigma\:}$ accounts for self-discharge losses. The converter operates with an efficiency of $\:{\eta\:}_{cnv}$. The total number of batteries is given by $\:{N}_{bat}$, and $\:{P}_{\mathrm{avg}}$ refers to the mean load demand in kW. The autonomy period is indicated by $\:AH\:$in hours, and $\:{C}_{\mathrm{bat}}$ represents the nominal battery capacity in kWh. The overall round-trip efficiency of the battery system is $\:{\eta\:}_{bat},\:$and the permissible depth of discharge is denoted as$\:\:DOD$.

**Table 7:** Generic 4 h 1 MW Li-Ion features^64^.

Characteristics	Values	Unit
Model	Generic 4 h 1 MW Li-Ion	-
Minimum SOC	0.0	%
Roundtrip efficiency	90	%
Nominal voltage	600	V
Nominal capacity	4.22	MWh
Nominal capacity	7.03	MAh
Lifetimes	15	Yr
Cost of Investment	500,000	$
Replacement cost	500,000	$
Operation and maintenance (O&M) cost	5,000	$

#### Diesel generator model

Diesel generators are used as backup units in hybrid power systems because renewable sources depend on environmental conditions, and batteries cannot handle high load demands. The amount of fuel consumed for electricity generation can be expressed as^35^:

14 $$F_d= (\mathrm{a}\: T_d + \mathrm{b}\: P_d)$$

where $\:{F}_{d}$ represents the diesel generator fuel consumption rate ($\:L/h$), *a* is the fuel intercept coefficient ($\:L/kWh$), *T*_*d*_ denotes the generator’s rated capacity (kW), *b* is the fuel slope coefficient (L/kWh), $\:{P}_{d}$ is the actual power output of the diesel generator (kW).

**Fig. 16:**
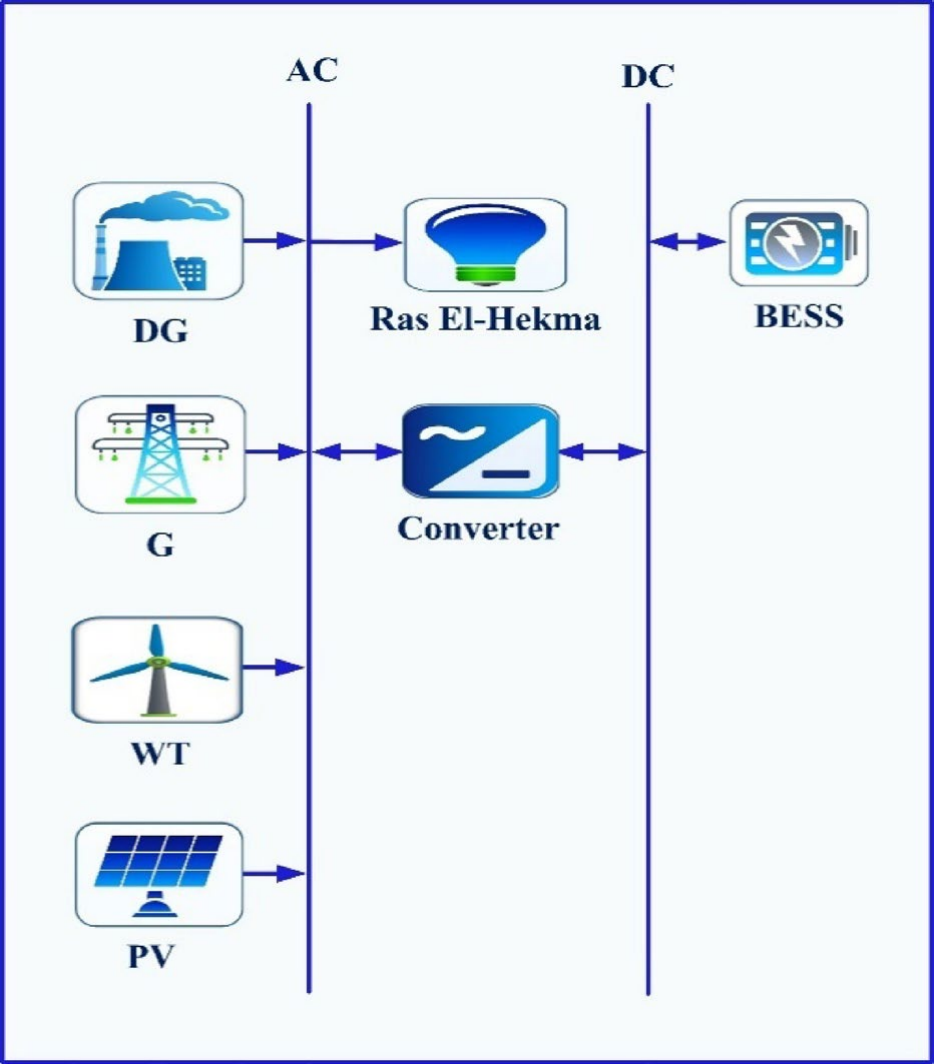
The arrangement of a hybrid power system.

The diesel generator adopted in the proposed HRES is based on the HOMER Pro Autosize Genset model, while the main technical and economic specifications of the DG are summarized in Table 8. In addition, the technical and economic parameters of the major system components considered in the HOMER Pro simulations are presented in Table 9. The overall configuration of the investigated hybrid renewable energy system is illustrated in Fig. 16.

**Table 8:** Autosize genset features^37^.

Characteristics	Value	Unit
Model	Autosize Genset	-
Fuel	Diesel	-
Fuel intercept curve	46.9	L/hr
Lifetime	15,000	Hr
Initial cost	250	$/kW
Replacement cost	250	$/kW
Operation and maintenance (O&M) cost	0.03	$/operating hour
Diesel fuel price	1.0	$/L

#### Grid

Electricity in Egypt is purchased from the utility grid at a grid power price of approximately $0.045/kWh, while the grid sellback price considered in this study is 0.04 $/kWh, according to the regulations of the Egypt ERA. In the grid-connected configurations, the grid functions as both a supplementary source and a sink for excess generation, compensating for shortfalls when renewable output is insufficient to satisfy demand while absorbing surplus power when production exceeds consumption. Generally, within the HOMER Pro tool, grid electricity purchase and excess electricity export are automatically incorporated into the economic calculations according to the adopted grid interaction settings and the internal HOMER Pro optimization framework. Consequently, differences between annual generation and demand may naturally appear in systems with high renewable energy penetration and/or battery storage due to the operational flexibility and energy management requirements considered during the optimization process.

**Table 9:** Specification of system components.

Item	Capacity	Investment cost	Replacement cost	Operating & Maintenance	Ref.
	(kW)	($)	($)	($)	
WT	1,000	1,650,000	1,600,000	40,000/yr	^54^
PV	20	3,000	3,000	10/yr	^56^
BESS	1,000	500,000	500,000	5000/yr	^64^
Converter	500	590	590	0.018/yr	^47^
DG	Autosize	250/kW	250/kW	0.03/op.hr	^37^

### Constraints and economics

The optimization process usually aims to identify the most feasible hybrid renewable energy system configuration based on technical, economic, and environmental criteria^17^. In HOMER Pro, the optimization procedure is performed by defining a set of allowable operational and economic constraints that govern the system design and simulation process. Based on these predefined settings, HOMER evaluates all possible configurations and determines the optimal solution according to the selected objective function. In this study, the primary objective function (OF) is the minimization of the COE, which can be expressed as^52^:

15 $$\:\mathrm{O}\mathrm{F}=min\left(\mathrm{C}\mathrm{O}\mathrm{E}\right)$$

subject to several technical and operational constraints. The generated renewable power ($\:{P}_{gen}$), exchanged grid power ($\:{P}_{grid}$), and battery power ($\:{P}_{bat}$) must satisfy the load demand ($\:{P}_{load}$) at all operating conditions according to:

16 $$\:{P}_{gen}+{P}_{grid}+{P}_{bat}\ge\:{P}_{load}$$

To guarantee both technical viability and operational dependability, a zero-percent maximum annual capacity shortage ($\:\mathrm{C}\mathrm{S}$) is enforced within HOMER Pro:

17 $$\:{\mathrm{C}\mathrm{S}}_{max}=0\%$$

thereby permitting only configurations capable of satisfying the entire yearly electrical demand^65^. This requirement is particularly important considering Ras El-Hekma’s planned role as a major center for urban expansion, tourism, and commercial activities, where supply interruptions are unacceptable.

To preserve design flexibility and accommodate Case 3 (the grid-only scenario), the minimum renewable fraction ($\:\mathrm{R}\mathrm{F}$) is constrained as:

18 $$\:\mathrm{R}\mathrm{F}\ge\:0$$

Operating reserves ($\:\mathrm{O}\mathrm{R}$) are introduced to mitigate demand variability and renewable output fluctuations as follows^19^:

19 $$\:\left\{\begin{array}{l}{\mathrm{O}\mathrm{R}}_{load}=10\% \\\:{\mathrm{O}\mathrm{R}}_{PV}=80\% \\\:{\mathrm{O}\mathrm{R}}_{WT}=50\%\end{array}\right.$$

In addition, the battery state of charge ($\:\mathrm{S}\mathrm{O}\mathrm{C}$) is maintained within the permissible operating range according to:

20 $$\:{\mathrm{S}\mathrm{O}\mathrm{C}}_{min}\le\:\mathrm{S}\mathrm{O}\mathrm{C}\le\:{\mathrm{S}\mathrm{O}\mathrm{C}}_{max}$$

The economic assessment incorporates key financial parameters that significantly influence project feasibility and long-term economic performance. The adopted discount and inflation rates are based on data reported by the Central Bank of Egypt. Within the HOMER Pro framework, these parameters are directly specified as economic inputs, while the corresponding real discount rate is internally calculated according to Eq. (3) and subsequently utilized during the economic assessment and optimization process. Based on the adopted economic parameters in this study, the resulting real discount rate is approximately 6.59%. At the same time, the project lifetime considered in this study is 20 years, as summarized in Table 10.

**Table 10:** Economics considered for hybrid model design.

Economic parameters	Value	Unit
Discount rate	24.5	%
Inflation rate	16.8	%
Project lifetimes	20.0	Yr

### Assumptions, limitations, and uncertainties of the study

To simplify the analysis in this study, the following assumptions are considered:

- The climatic data used in the simulations are assumed to be representative of the long-term conditions of the Ras El-Hekma study area.
- The technical characteristics of the system components are assumed constant throughout the project lifetime according to the adopted HOMER Pro database and manufacturers’ specifications.
- The system components are assumed to operate under normal operating conditions without unexpected failures or shutdowns.
- The degradation effects of PV modules, wind turbines, batteries, and converters are neglected during the simulation period.
- The economic parameters, including inflation rate, discount rate, and fuel price are assumed constant during the project lifetime.
- The grid electricity purchase and sellback prices are assumed constant during the simulation period according to the adopted regulated electricity tariff structure to ensure a consistent comparative techno-economic assessment framework.

The principal limitation of this study is that the analysis is based on historical climatic and economic data, while future variations in renewable resources, economic conditions, and energy market prices may affect the actual system performance and economic feasibility. In addition, the HOMER Pro analysis is based on hourly energy balance simulations and built-in optimization strategies under predefined technical and economic assumptions. Furthermore, the environmental assessment within the HOMER Pro framework primarily considers operational emissions during the system operation stage, while detailed lifecycle environmental impacts associated with the manufacturing and disposal of system components are not explicitly modeled.

The primary sources of uncertainty are related to climatic variability, future economic fluctuations, component cost variations, and the assumptions adopted during the simulation and optimization processes. In particular, the availability of renewable energy resources, such as solar irradiance and wind speed, is strongly influenced by weather conditions, which may impact the generated power and overall system performance^52^. In addition, future changes in component prices and economic indicators may influence the techno-economic assessment results.

## Results and discussions

In this section, a detailed assessment of the six energy system configurations is presented for the Ras El-Hekma development area. Using HOMER Pro as the simulation platform, each configuration is evaluated across multiple aspects, including technical performance, economic viability, environmental consequences, and sensitivity to variations in critical financial variables. Table 11 shows the optimization results for the hybrid power model with and without a grid connection, respectively.

**Table 11:** The optimal design of the six cases.

Case No.	Architecture						Cost			
	PV	DG	G	Converter	WT	BESS	NPC	COE	Operating cost	Initial cost
	kW				quantity		$	$/kWh	$/yr	$
1	-	-	On	-	15	-	37.5 M	0.0341	1.16 M	24.8 M
2	-	-	On	-	12	1	38.2 M	0.0383	1.63 M	20.3 M
3	-	-	On	-	-	-	39.0 M	0.0450	3.56 M	0
4	-	-	-	30,804	35	138	189 M	0.2190	4.25 M	143 M
5	263	-	-	34,395	34	144	195 M	0.2250	4.40 M	147 M
6	27.2	32,000	-	30,876	34	83	185 M	0.2130	2.50 M	122 M

### Economic analysis

**Fig. 17:**
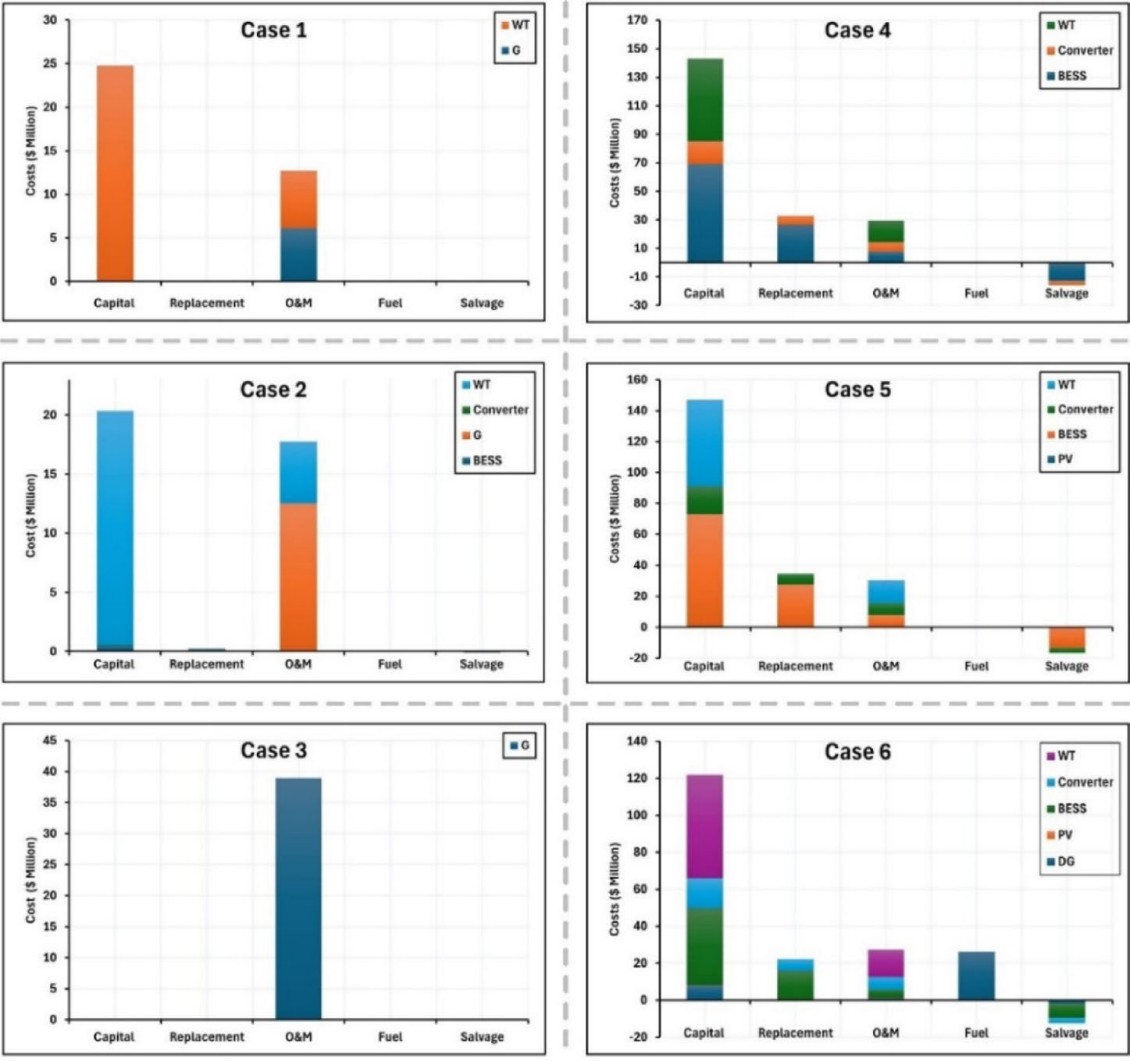
Cost comparisons.

**Fig. 18:**
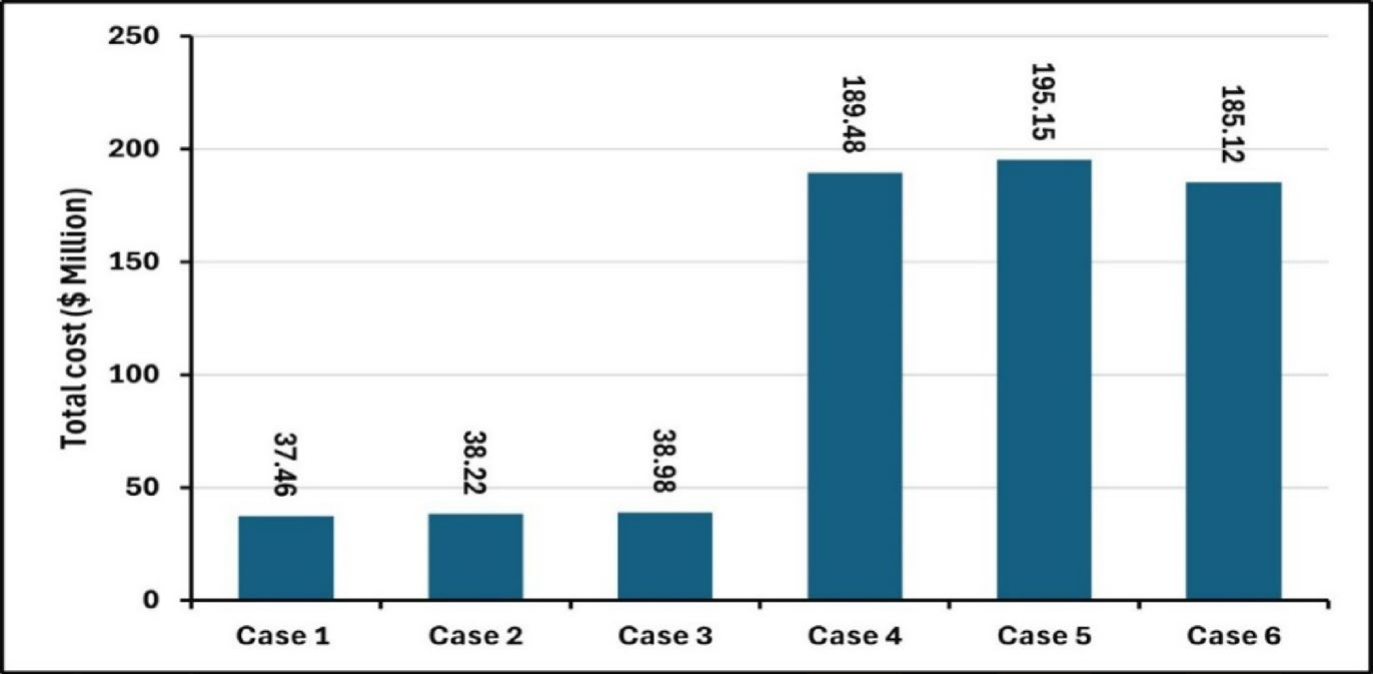
Total cost of all cases.

The overall cost results of the six cases are presented in Table 12. It is notable that among the different cases evaluated, Case 1 (WT + G) has the most cost-effective design. It achieves the lowest COE, at $0.03409/kWh, and the lowest total NPC, at $37.46 million, as well as the lowest operating and maintenance (O&M) cost of around $12.71 million. Although Case 3 (G) requires no upfront capital investment, its highest O&M costs lead to a higher long-term cost, with a COE of about $0.04495/kWh and a total NPC of $38.98 million. Case 1 is also more economical than Cases 4, 5, and 6, which have significantly higher capital costs due to heavy reliance on BESS. However, the RF of Case 1 is only 68.6%, which is much lower than the 100% RF achieved in Cases 4 and 5, as well as in Case 6. Although Case 6 reduces the initial capital cost by using a diesel generator, it also results in higher O&M costs and increased emissions, making it less favorable both economically and environmentally. While Case 2 (WT + BESS + G) has a slightly higher NPC compared to Case 1, it provides greater reliability by combining wind power, battery storage, and grid support more effectively. Figure 17 visualizes the cost comparison, showing various costs such as Capital, Replacement, O&M, fuel, and Salvage, while the total cost for all cases studied is summarized in Fig. 18.

**Table 12:** Overall cost results of HOMER.

System	Capital	NPC	COE	O&M cost	RF %
	($)	($)	($/kWh)	($)	
Case 1	24,750,000	37,458,100	0.03409	12,708,097.41	68.6
Case 2	20,345,550.39	38,217,120	0.03831	17,763,630.88	60.6
Case 3	0	38,976,370	0.04495	38,976,366.98	0.00
Case 4	143,014,288.46	189,483,400	0.21870	29,599,109.30	100
Case 5	147,049,568.82	195,148,400	0.22520	30,304,143.96	100
Case 6	121,983,857.68	185,120,100	0.21350	27,348,395.97	98.7

### Environmental impact

**Fig. 19:**
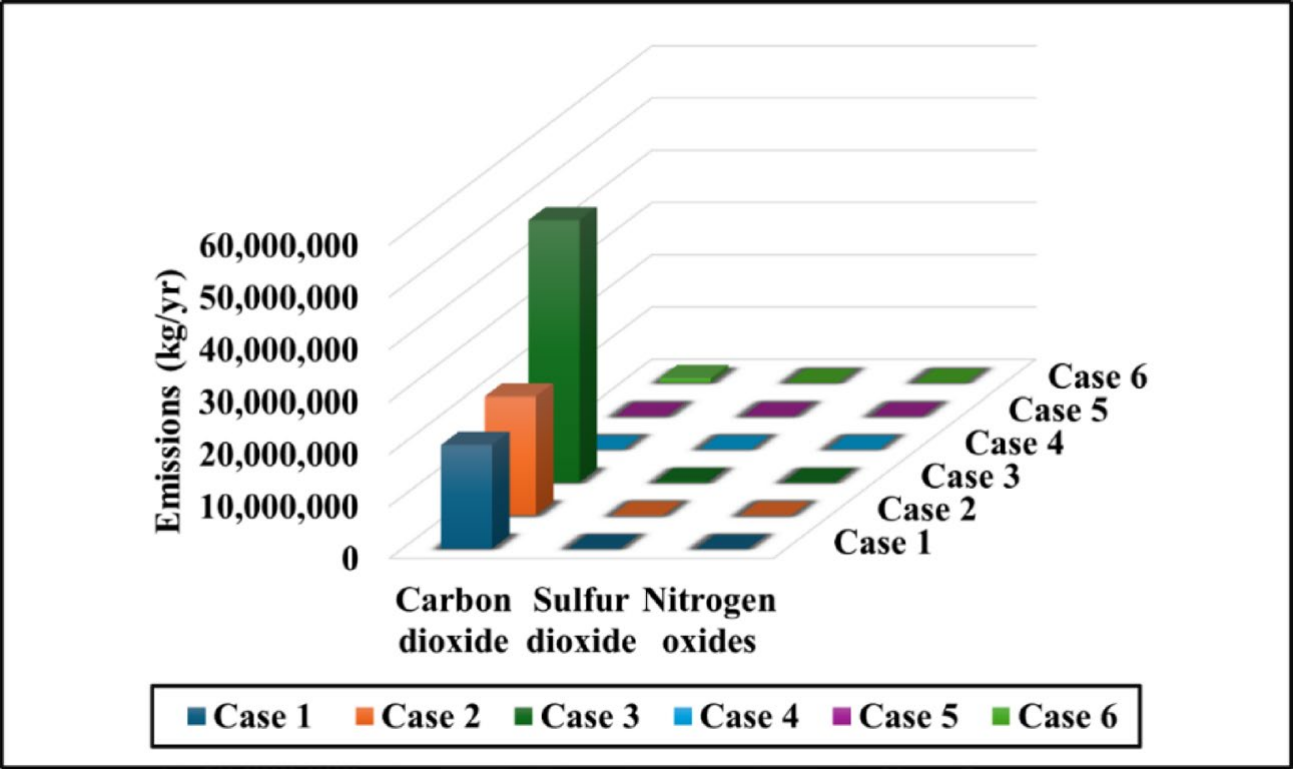
Annual CO₂, SO₂, and NOx emissions for each configuration.

The transition toward HRES plays a significant role in reducing CO₂ emissions and mitigating the environmental impacts associated with conventional fossil-fuel-based electricity generation^66^. In this study, the environmental impact assessment focuses on annual emissions of CO_2_, SO_2_, and NOx for each configuration, as illustrated in Table 13; Fig. 19. The results show that Case 1 and Case 2 reduced CO₂ emissions by 60.452% and 54.704%, respectively, compared to the base case (Case 3). These two cases are therefore well-suited for projects aiming to balance cost-effectiveness with environmental impact mitigation. Since Cases 4 and 5 operate solely on renewable energy, they produce no operational emissions. However, their high initial costs limit their practical adoption. Case 6 records moderate emissions due to the limited use of a diesel generator. In contrast, Case 3 demonstrates the highest emission levels because of its dependence on natural gas, making it the least favorable scenario from an environmental perspective.

**Table 13:** Emission comparisons.

System	Carbon dioxide	Sulfur dioxide	Nitrogen oxides
	(kg/yr)	(kg/yr)	(kg/yr)
Case 1	19,813,948	85,902	42,011
Case 2	22,693,959	98,388	48,117
Case 3	50,101,343	217,212	106,228
Case 4	0	0	0
Case 5	0	0	0
Case 6	785,366	1,923	4,650

### Energy production and consumption analysis

Energy production and distribution for all configurations are summarized in Tables 14 and 15. Case 1 generates approximately 100.5 GWh/year, while the local demand is around 79 GWh per year, and the remaining energy is exported to the national grid. Similarly, Case 2 (WT + BESS + G) produces slightly less, about 91.2 GWh/year, and its excess energy is also exported. For the off-grid configurations (Cases 4–6), maintaining a 0% unmet load results in a substantial surplus, ranging from 47% to 51% of total generation, as the system must continuously guarantee load supply under intermittent renewable resource conditions. Excess electricity refers to the portion of generated energy that exceeds the load demand when renewable sources produce more power than is needed, and the battery storage is already at full capacity^67^. This surplus can significantly affect the system’s reliability, stability, and overall efficiency, making it a critical consideration in the design and operation of off-grid hybrid renewable energy systems^68,69^. It should also be noted that HOMER Pro evaluates a broad range of feasible system combinations under predefined technical and economic constraints. Consequently, some off-grid configurations may exhibit relatively high excess electricity levels as part of the optimization search space, particularly under strict reliability requirements. Such behavior represents a common reliability–efficiency trade-off in standalone renewable energy systems rather than a modeling inconsistency. Therefore, the occurrence of high excess electricity in certain configurations should be interpreted within the context of ensuring uninterrupted power supply under varying renewable resource conditions. In contrast, Case 3 (the base case) relies entirely on the grid to meet local demand, which results in higher carbon emissions.

**Table 14:** Comparison of annual electrical production.

System	WT	PV	DG	Grid purchases	Total
	(kWh/yr)	(kWh/yr)	(kWh/yr)	(kWh/yr)	(kWh/yr)
Case 1	69,108,331	0	0	31,351,184	100,459,515
Case 2	55,286,665	0	0	35,908,163	91,194,828
Case 3	0	0	0	79,274,277	79,274,277
Case 4	161,252,773	0	0	0	161,252,773
Case 5	156,645,550	81,362	0	0	156,726,912
Case 6	156,645,550	47,687	1,048,660	0	157,741,898

**Table 15:** Comparison of annual consumption and sales.

System	AC primary load	Grid sales	Total	Excess energy	Excess energy	Unmet load
	(kWh/yr)	(kWh/yr)	(kWh/yr)	(kWh/yr)	(%)	(%)
Case 1	79,274,277	21,185,238	100,459,515	0	0	0
Case 2	79,274,277	11,919,340	91,193,617	1,812	0.002	0
Case 3	79,274,277	0	79,274,277	0	0	0
Case 4	79,223,573	0	79,223,573	77,653,507	48.2	0.064
Case 5	79,232,195	0	79,232,195	73,453,076	46.9	0.0531
Case 6	79,274,277	0	79,274,277	74,214,883	47	0

**Fig. 20:**
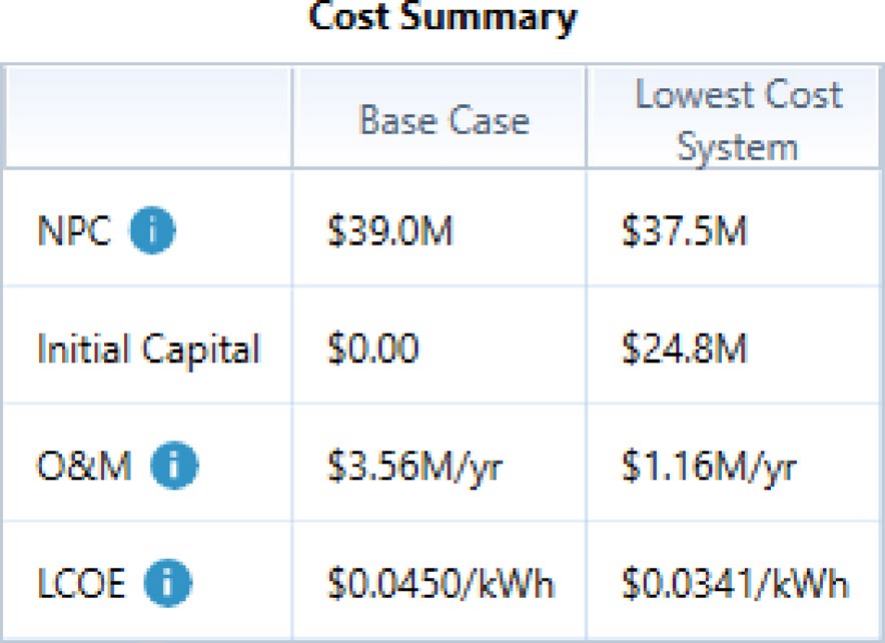
Summary cost of the base case (case 3) and case 1.

**Fig. 21:**
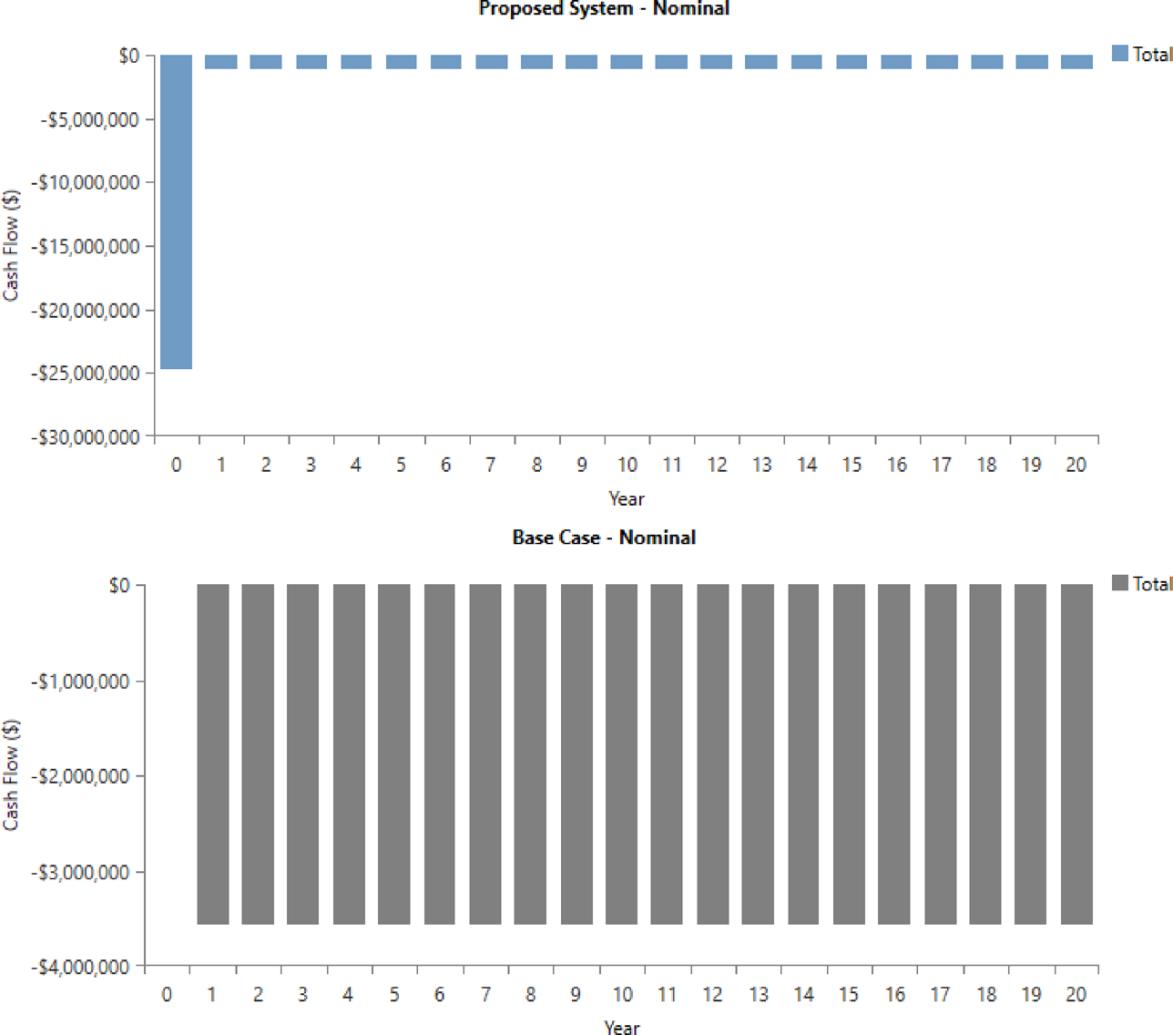
Economy comparison of base and proposed cases.

Figures 20 and 21 illustrate a significant reduction in both operating costs and environmental impact in Case 1 compared to Case 3, which relied solely on grid electricity. Although Case 3 requires no capital investment, its high long-term operating costs made it less favorable in terms of NPC. In contrast, despite the higher initial investment, Case 1 achieves a significantly lower NPC, demonstrating the long-term cost-effectiveness of integrating renewable energy sources.

**Fig. 22:**
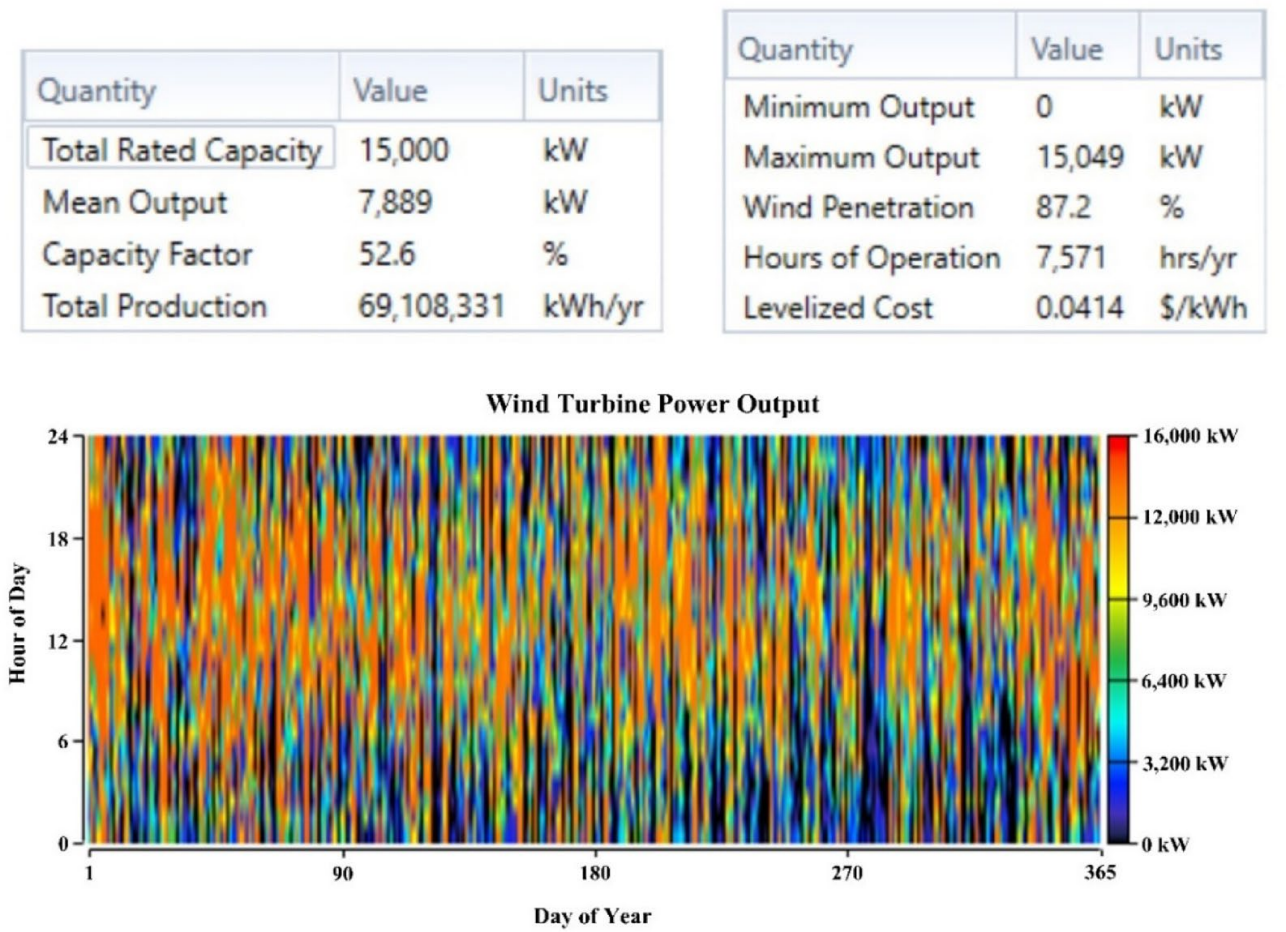
The electrical energy generation of the wind turbine system over the year.

**Fig. 23:**
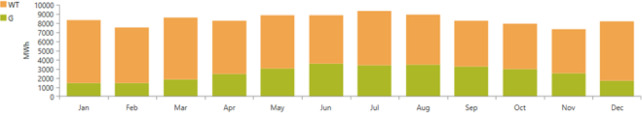
The monthly electricity production of case 1 for 12 months.

**Fig. 24:**
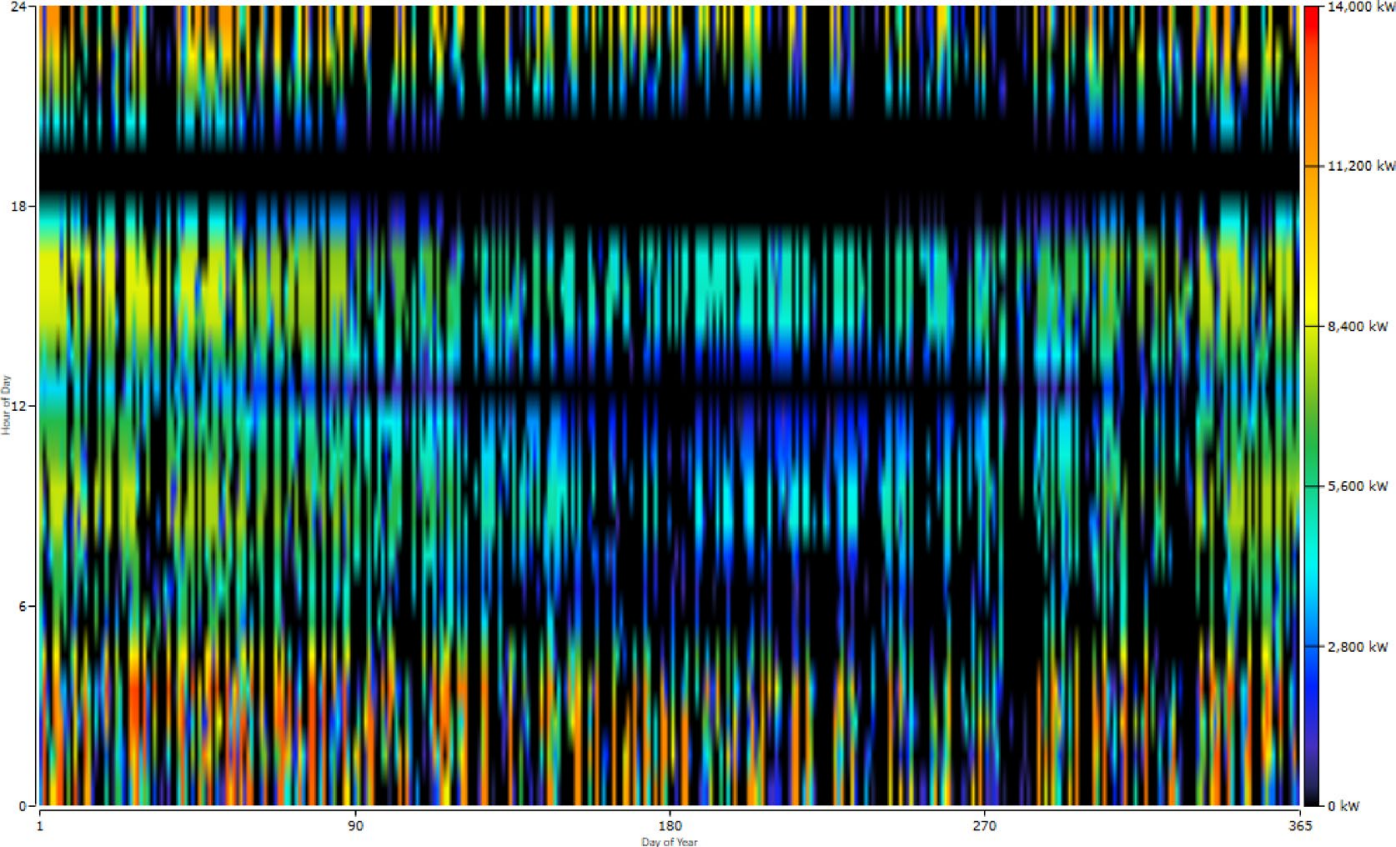
Grid sales data map.

**Fig. 25:**
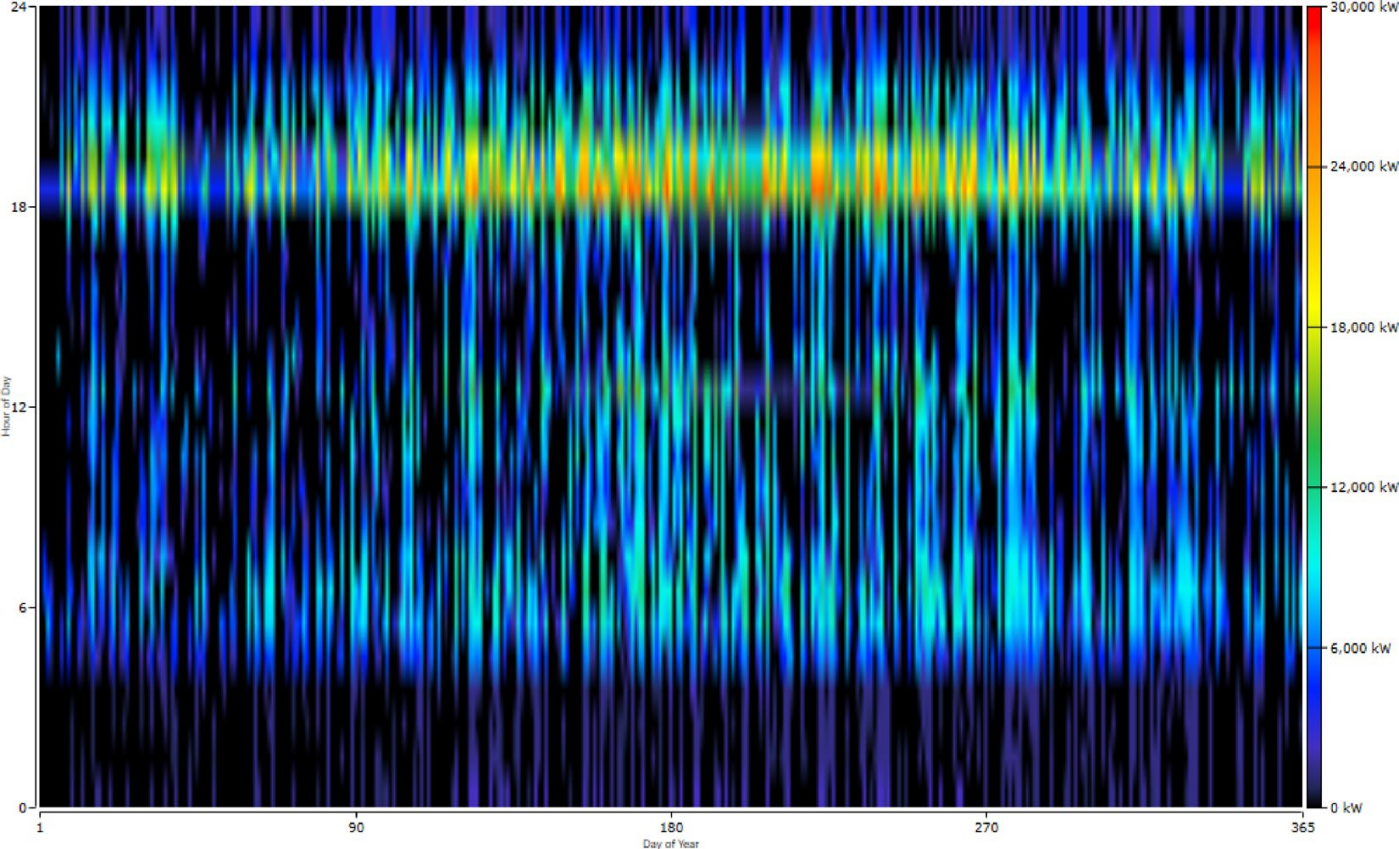
The grid energy purchase map over the year.

Case 1 (WT + G) represents the most financially favorable configuration, providing a minimal COE while also yielding considerable environmental benefits. This system primarily relies on the wind turbine to meet the local load. During periods when the wind turbine cannot fully meet demand, particularly between 18:00 and 19:00, additional energy is purchased from the grid. Conversely, during periods of high wind generation, surplus energy is exported to the grid, generating revenue that partially offsets the cost of the purchased electricity. The 15 MW wind turbine produces 69,108.3 MWh annually, with a mean output of 7.9 MW and a high-capacity factor of 52.6%, reflecting efficient operation and strong wind potential at the site. The electrical energy generation of the wind turbine system over the year is shown in Fig. 22. The monthly electricity production of case 1 is presented in Fig. 23. Grid interactions are important for the system’s economics, with around 21.2 GWh per year exported to the grid, as illustrated in Fig. 24, while 31.4 GWh/year is purchased during low-wind periods. Figure 25 presents the grid energy purchase map over the year.

### Sensitivity analysis

This section presents a detailed sensitivity analysis examining how uncontrollable input parameters, including the discount rate, inflation rate, and project lifespan, influence the selection and performance of optimal system configurations. It should be noted that, within the HOMER Pro tool, environmental indicators are directly linked to the operational behavior and energy mix of each investigated configuration. Therefore, the environmental impact is inherently reflected through the comparative environmental assessment presented in Sect. 4.2, while the present sensitivity analysis focuses primarily on critical techno-economic parameters affecting the system decision-making process.

#### Sensitivity analysis of average wind speed on optimal system parameters

The power generated by a wind turbine is largely determined by the prevailing wind speed at the installation site^70,71^. This section examines how fluctuations in mean wind velocity affect the choice of the most suitable wind farm configuration for the study area. Economic performance is assessed using total NPC and COE, while environmental implications are quantified in terms of carbon dioxide (CO₂) emissions.

**Fig. 26:**
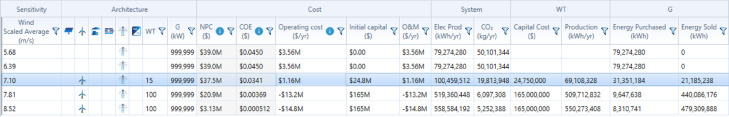
Impact of the variation of average wind speed on the parameter of the system.

**Fig. 27:**
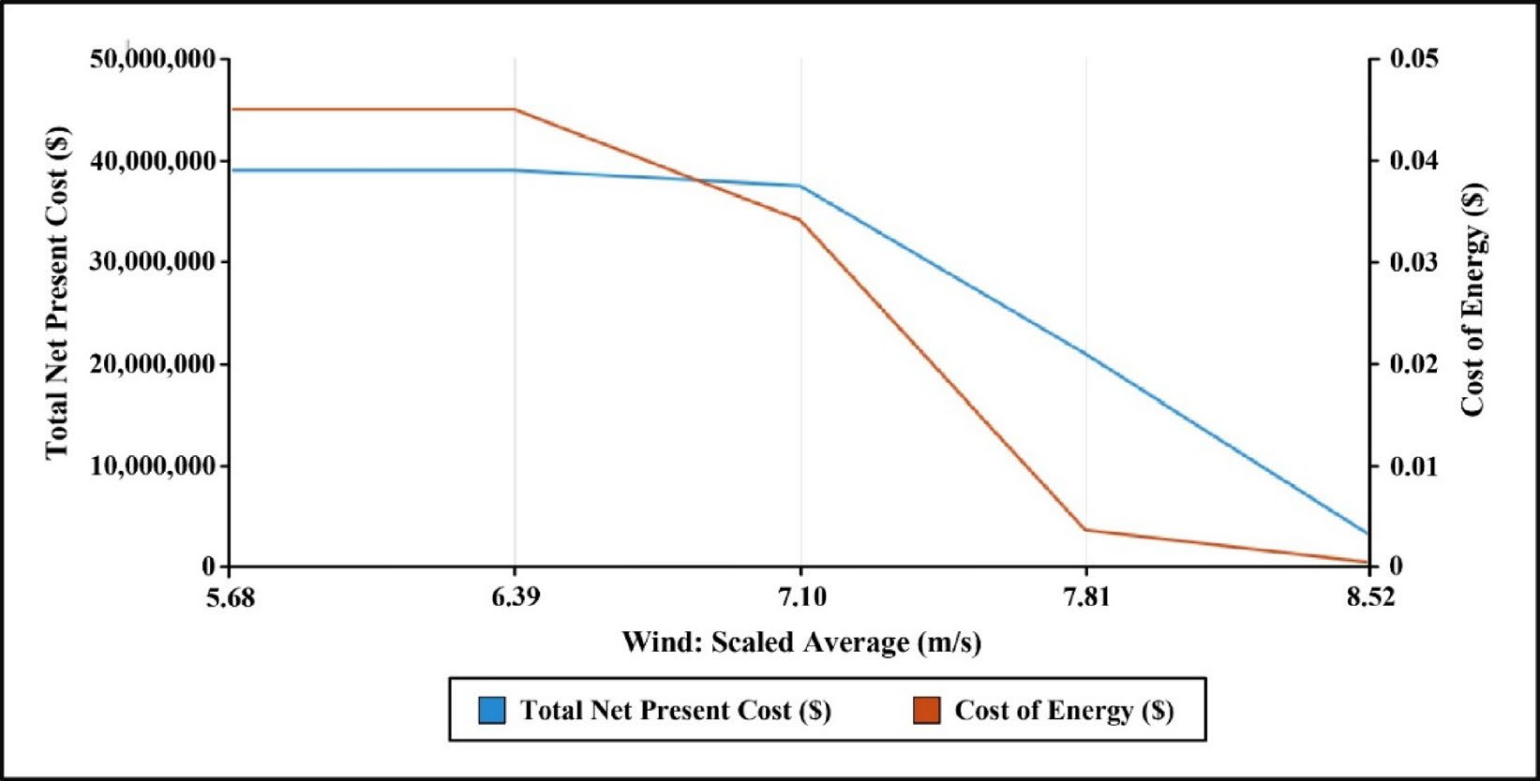
Impact of wind speed on total NPC and COE.

In the sensitivity analysis, the average wind speed is varied by ± 10% and ± 20% relative to the base-case value of 7.10 m/s. The results show that higher wind speeds lead to the inclusion of more wind turbine units in the optimal scenario, while TNPC, COE, and carbon dioxide emissions decrease, as illustrated in Figs. 26 and 27. The obtained sensitivity-analysis trends are also consistent with previously published HOMER Pro-based studies. Specifically, the observed reduction in TNPC, COE, and CO₂ emissions with increasing average wind speed, together with the increased integration of wind turbines within the optimal configuration, aligns with the findings reported in the literature^72^. This agreement further supports the consistency and credibility of the obtained techno-economic assessment results.

#### Sensitivity analysis of the normal discount rate on cost parameters

**Fig. 28:**
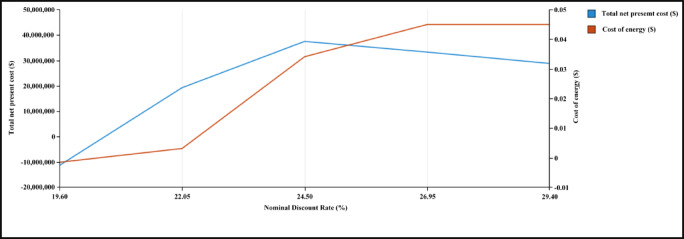
Impact of discount rate on total NPC and COE.

Investment choices in Egypt are significantly shaped by the prevailing nominal discount rate. To thoroughly evaluate how this factor influences system costs, a sensitivity analysis is performed across five distinct discount rate scenarios, which are the baseline rate, along with adjustments of ± 10% and ± 20%. As illustrated in Fig. 28, the outcomes align with the principle of the time value of money. When the discount rate is reduced, future cash flows carry more weight, thereby increasing present costs. In contrast, a higher discount rate prioritizes near-term cash flows, which correspondingly diminishes present costs^73^.

#### Sensitivity analysis of project lifetimes on cost parameters

**Fig. 29:**
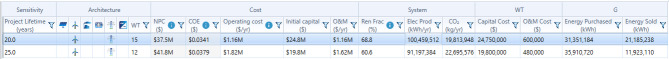
Impact of lifetimes on system parameters.

**Fig. 30:**
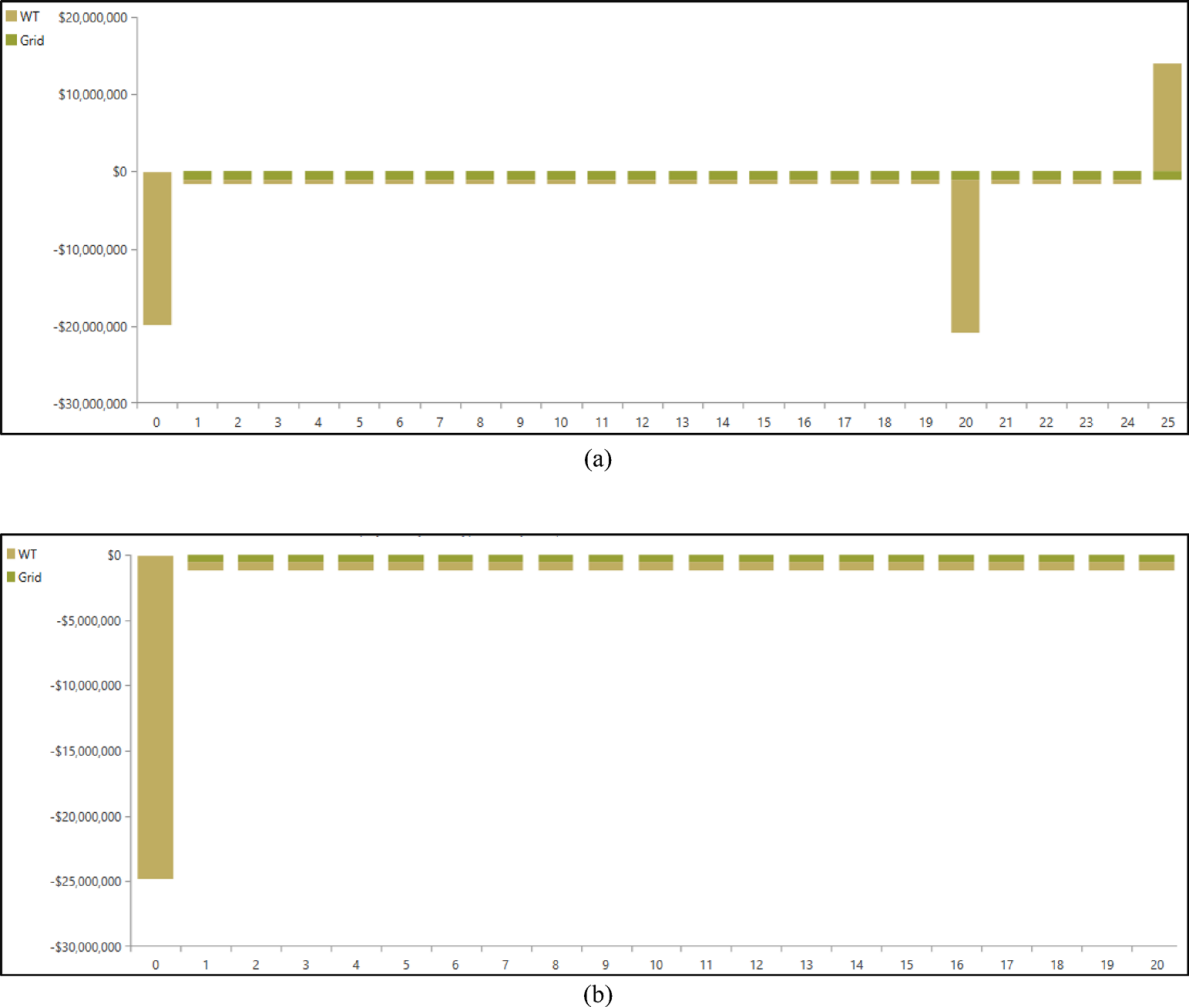
Cash flow of the (WT + G) configuration with two different lifetimes: **(a)** 25 years **(b)** 20 years.

An evaluation of 20-year and 25-year project lifetimes is undertaken to assess whether the optimized HRES configuration would yield superior cost-effectiveness and operational reliability. The results shown in Fig. 29 illustrate that the optimal project lifetime is 20 years, as it aligns with the lifespan of the selected wind turbines. In the case of a 25-year project lifetime, a replacement cost of $5,355,142.79 would be incurred in the 20th year to replace the wind turbines, as shown in Fig. 30. Therefore, the 20-year lifetime is more cost-effective compared to extending the project to 25 years.

#### Sensitivity analysis of inflation rates on cost parameters

**Fig. 31:**
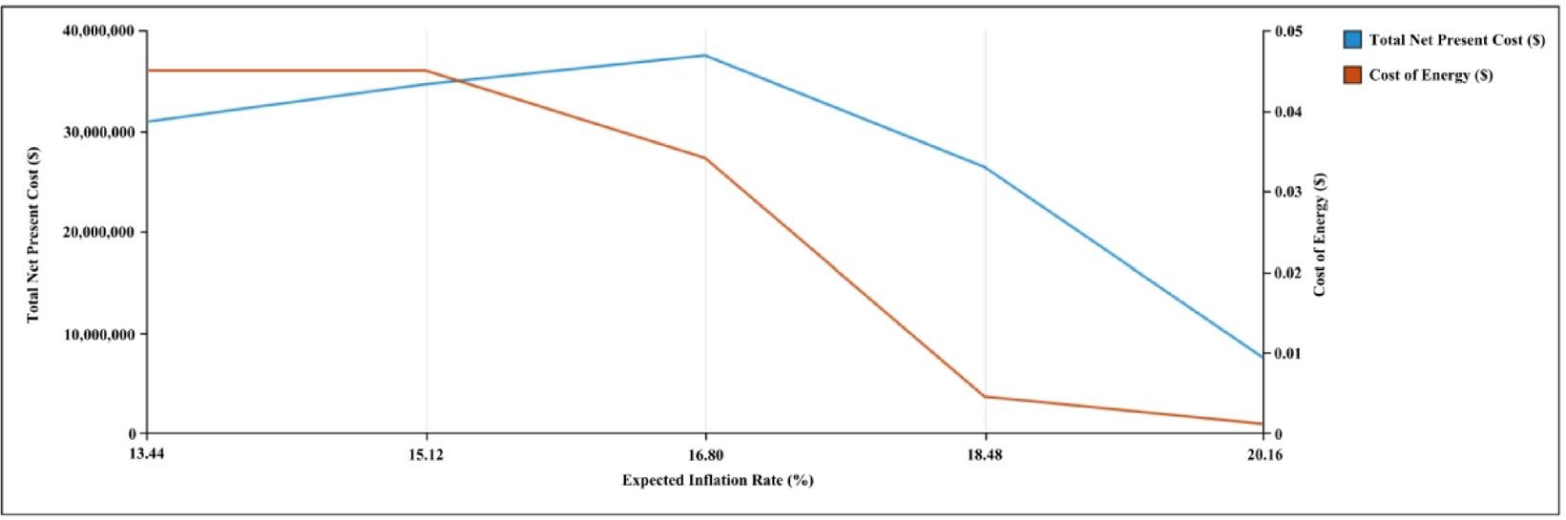
Impact of Inflation rates on cost parameters.

**Fig. 32:**
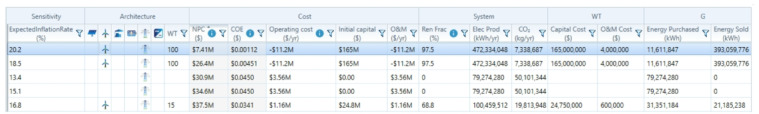
Impact of Inflation rates on system parameters.

The influence of inflation on system costs is examined through a sensitivity analysis employing five distinct inflation rate scenarios, which are the baseline rate, together with deviations of ± 10% and ± 20%. As illustrated in Figs. 31 and 32, the findings indicate that higher inflation rates correspond to a reduction in the COE.

#### Sensitivity analysis of both inflation and discount rates

**Fig. 33:**
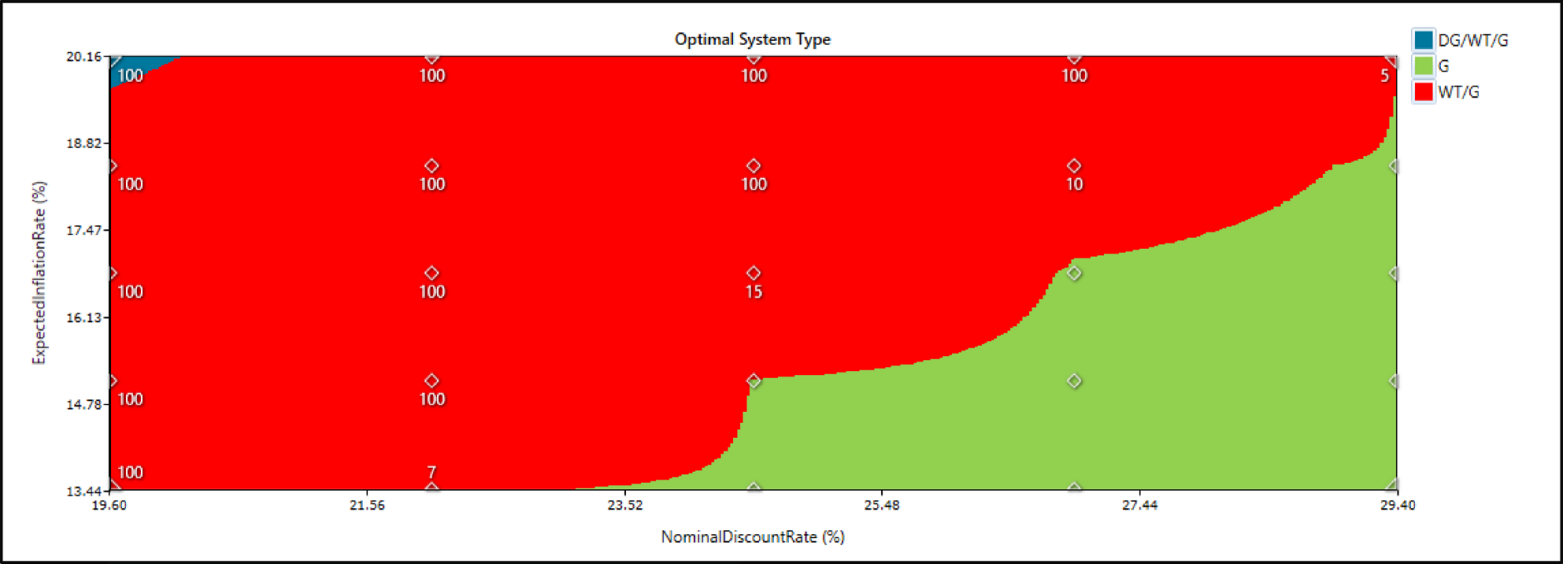
Impact of inflation and discount rate variations on optimal system configuration selection, superimposed with WT quantity.

**Fig. 34:**
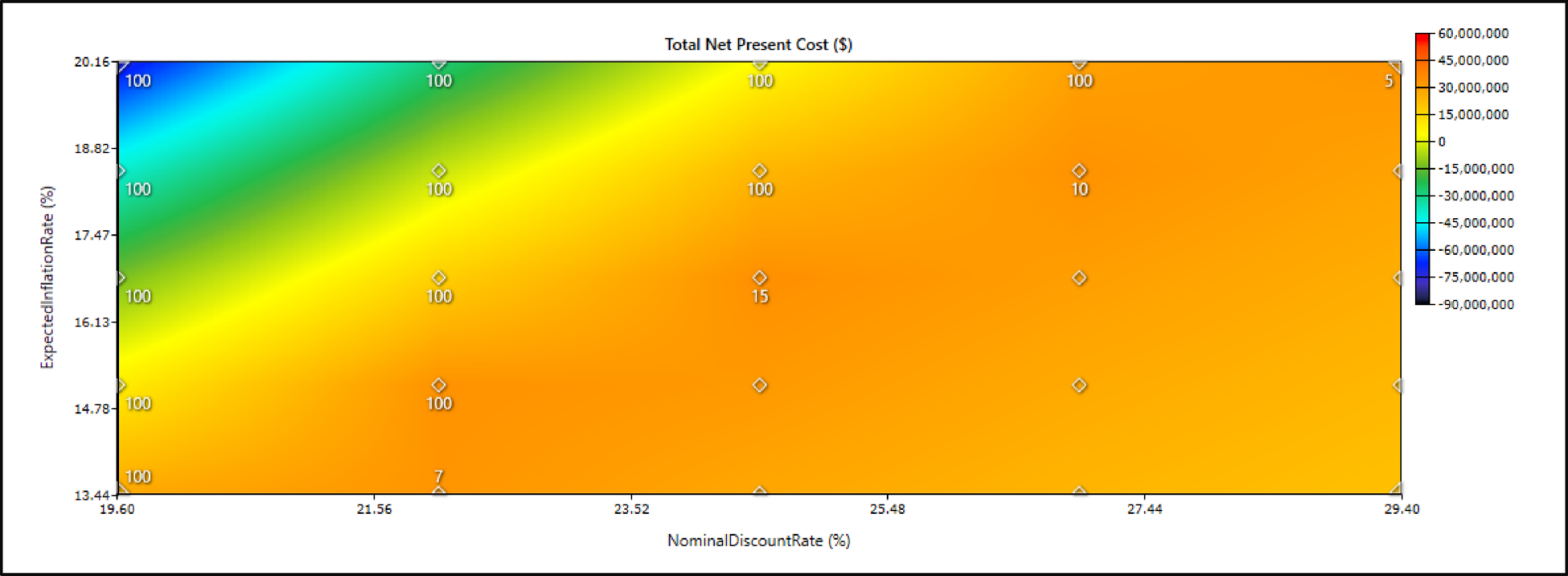
Impact of inflation and discount rate variations on total NPC, superimposed with WT quantity.

**Fig. 35:**
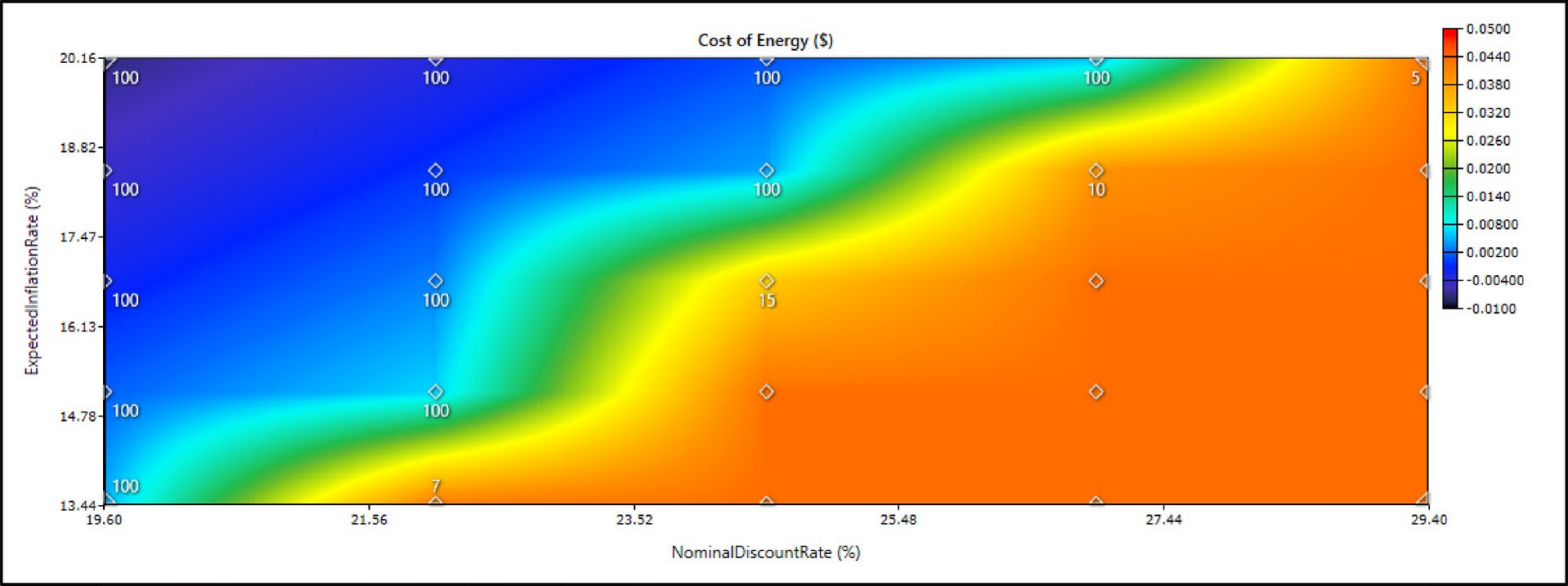
Impact of inflation and discount rate variations on the COE, superimposed with WT quantity.

A sensitivity analysis is performed to evaluate the impact of variations in inflation and discount rates on the selection of the optimal system configuration, as shown in Fig. 33, considering five different values for each parameter. The results show that, despite these variations, the wind turbine (WT) with grid connection consistently remains the optimal system type. Figures 34 and 35 illustrate how changes in inflation and discount rates affect the total NPC and the COE, respectively. Specifically, varying the interest rate from 19.60% to 29.40% and the discount rate from 13.44% to 20.16% increases the COE by 87.58% while decreasing the TNPC by 13.97%, indicating that these financial parameters have a significant effect on project economics.

## Conclusion

This study presented a techno-economic and environmental assessment of large-scale hybrid renewable energy systems for the Ras El-Hekma coastal megaproject in Egypt using HOMER Pro. The results demonstrated that the grid-connected WT/G configuration achieved the optimal overall performance with a COE of $0.03409/kWh, an NPC of $37.46 million, and a renewable energy fraction of 68.6%, while reducing CO₂ emissions by approximately 60.45% compared with the base case. In contrast, the off-grid configurations achieved higher renewable energy penetration and improved energy autonomy; however, they exhibited excess electricity levels of approximately 50% due to strict reliability requirements and the absence of grid support. Furthermore, the sensitivity analysis confirmed that wind speed, project lifetime, inflation rate, and discount rate significantly influence the economic feasibility and optimal system configuration. In particular, increasing the average wind speed resulted in lower NPC, COE, and CO_2_ emissions, together with higher wind turbine penetration in the optimal configurations. Future work may include advanced optimization frameworks with user-defined objective functions, validation using alternative optimization tools, improved coastal wind modeling, enhanced wind turbine selection methodologies, demand-side management strategies, detailed reliability assessment, and probabilistic uncertainty analysis.

## Data Availability

All data generated or analysed during this study are included in this published article.

## Abbreviations

WT: Wind Turbine
PV: Photovoltaic
DG: Diesel Generator
BESS: Battery Energy Storage System
G: Grid
PHES: Pumed Hydro Energy Storage
BIOMG: Biomass Generator
HRES: Hybrid Renewable Energy System
RESs: Renewable Energy Sources
$: United States Dollar
NPC: Net Present Cost
COE: Cost of Energy
RF: Renewable Fraction
DNI: Direct Normal Irradiance
GHI: Global Horizontal Irradiance
DHI: Diffuse Horizontal Irradiance
NASA: Aeronautics and Space Administration
OC: Operating cost
O&M: Operation and Maintenance
FC: Fuel Cost
CC: Capital Cost
RC: Replacement Cost
SV: Salvage Value
STC: Standard Test Condition

## References

[1] Abo-Khalil, A. G., Sobhy, A., Abdelkareem, M. A. & Olabi, A. G. Advancements and challenges in hybrid energy storage systems: Components, control strategies, and future directions. *Int. J. Thermofluids*. **20**, 100477 (2023).

[2] Elfar, M. H. et al. Optimal parameters identification for PEMFC using autonomous groups particle swarm optimization algorithm. *Int. J. Hydrog Energy*. **69**, 1113–1128 (2024).

[3] Abo-Khalil, A. G., Lei, D. & Sobhy, A. Nonlinear auto disturbance rejection control for DFIG-based wind turbines under unbalanced grid voltage circumstances. *Results Eng.* **27**, 106271 (2025).

[4] Al-Maghalseh, M. The Environmental Impact and Societal Conditions of PV Power Plants: A Case Study of Jericho Gate-Palestine Stat Of. *Wadi Alshatti Univ. J. Pure Appl. Sci.* **3** (2), 16–31 (2025).

[5] Amhimmid, A. A. et al. Financial Modeling of Social and Environmental Impacts of Wind Farm in Urban Zones: A Case Study of Zawia-Libya. *Int J. Energy Environ. Eng* **15**(4), 1–19 (2024).

[6] Nassar, Y. F. et al. Technical and environmental cost-benefit analysis of strategies towards a green economy in the electricity sector in Libya. *Econ. Policy Energy Environ.* **2**, 133–167 (2025).

[7] Abuhelwa, M. et al. Exploring the Prevalence of Renewable Energy Practices and Awareness Levels in Palestine. *Energy Sci. Eng.* **13**, 1292–1305 (2025).

[8] Abdallah, L. & El-Shennawy, T. *Future of Renewable Energy in Egypt: Challenges and Opportunities*. (2022).

[9] Obukhov, S. & Ibrahim, A. Analysis of the Energy Potential of Renewable Energy Sources Egypt. *MATEC Web Conf.* **141**, 01035 (2017).

[10] Patlitzianas, K. D. Solar energy in Egypt: Significant business opportunities. *Renew. Energy*. **36**, 2305–2311 (2011).

[11] Moharram, N. A., Tarek, A., Gaber, M. & Bayoumi, S. Brief review on Egypt’s renewable energy current status and future vision. *Technol. Mater. Renew. Energy Environ. Sustain.* **8**, 165–172 (2022).

[12] Gibson, A. et al. Long-Term Energy System Modelling for a Clean Energy Transition in Egypt’s Energy Sector. *Energies* **17**, 2397 (2024).

[13] Basiouny, M., Kafrawy, S., Ghanem, E. & Soliman, A. Shoreline Change Rate Detеction and Futurе Prediction Using Rеmote Sensing and GIS Tеchniques: A Case Study of Ras EL-Hekma, North Western Coast, Egypt. *J. Geogr. Environ. Earth Sci. Int.* **9**, 1–1432086 (2017).

[14] Oraby, M. A., Marmoush, R. Y., El-Badry, H. M. & Abdelsalheen, M. H. The shoreline and morphological responses to storm event at Ras Al-Hekma sandy beaches. *Ain Shams Eng. J.* **15**, 103132 (2024).

[15] Mohammad Husain, A., Muzaffarul Hasan, M., Khan, Z. A. & Asjad, M. A robust decision-making approach for the selection of an optimal renewable energy source in India. *Energy Convers. Manag*. **301**, 117989 (2024).

[16] Hassan, Q., Algburi, S., Sameen, A. Z., Salman, H. M. & Jaszczur, M. A review of hybrid renewable energy systems: Solar and wind-powered solutions: Challenges, opportunities, and policy implications. *Results Eng.* **20**, 101621 (2023).

[17] Abduallah, A. et al. Leveraging Hydrogen for Covering Energy Shortage in an Electricity Subgrid. *Wadi Alshatti Univ. J. Pure Appl. Sci.* **4** (1), 245–254 (2026).

[18] Alfathi, S. A., Miskeen, G. & Mremi, W. Evaluation and Prediction Performance of Solar Panel and Wind Turbine Systems Using Simulation. *Wadi Alshatti Univ. J. Pure Appl. Sci.* **4** (1), 94–104 (2026).

[19] Abduallah, A. et al. Integrating Electricity Sub-Grid with Pumped Hydropower Storage System for Grid Stability and Sustainability. *Wadi Alshatti Univ. J. Pure Appl. Sci.* **3** (2), 322–332 (2025).

[20] Nassar, Y. F. et al. Design of reliable standalone utility-scale pumped hydroelectric storage powered by PV/Wind hybrid renewable system. *Energy Convers. Manag*. **322**, 119173 (2024).

[21] El-Khozondar, H. J. et al. Technical-economical-environmental assessment of grid-connected hybrid renewable energy power system for Gaza Strip-palestine. *Eng. Sci. Technol. Int. J.* **69**, 102120 (2025).

[22] Nassar, Y. F., El-Khozondar, H. J. & Fakher, M. A. The role of hybrid renewable energy systems in covering power shortages in public electricity grid: An economic, environmental and technical optimization analysis. *J. Energy Storage*. **108**, 115224 (2025).

[23] Bahgaat, N. K. Estimation of renewable energy systems for mobile network based on real measurements using HOMER software in Egypt. *Sci. Rep.* **13**, 16713 (2023). 37794103 10.1038/s41598-023-43877-2 PMC10550915

[24] Hoarcă, I. C., Bizon, N., Șorlei, I. S. & Thounthong, P. Sizing Design for a Hybrid Renewable Power System Using HOMER and iHOGA Simulators. *Energies* 16, (2023).

[25] Vargas-Salgado, C. et al. Validations of HOMER and SAM tools in predicting energy flows and economic analysis for renewable systems: Comparison to a real-world system result. *Sustain. Energy Technol. Assess.* **69**, 103896 (2024).

[26] Owolabi, A. B. et al. Validating the techno-economic and environmental sustainability of solar PV technology in Nigeria using RETScreen Experts to assess its viability. *Sustain. Energy Technol. Assess.* **36**, 100542 (2019).

[27] Giedraityte, A., Rimkevicius, S., Marciukaitis, M., Radziukynas, V. & Bakas, R. Hybrid Renewable Energy Systems—A Review of Optimization Approaches and Future Challenges. *Appl. Sci.* **15**, 1744 (2025).

[28] Ashetehe, A. A., Shewarega, F., Bantyirga, B., Biru, G. & Lakeo, S. Optimal design of off-grid hybrid system using a new zebra optimization and stochastic load profile. *Sci. Rep.* **14**, 29255 (2024). 39587285 10.1038/s41598-024-80558-0 PMC11589698

[29] Bahgaat, N. K. Estimation of renewable energy systems for mobile network based on real measurements using HOMER software in Egypt. *Sci. Rep.* **13**, 16713 (2023). 37794103 10.1038/s41598-023-43877-2 PMC10550915

[30] Chisale, S. W., Eliya, S. & Taulo, J. Optimization and design of hybrid power system using HOMER pro and integrated CRITIC-PROMETHEE II approaches. *Green Technologies and Sustainability* **1**, 100005 (2023).

[31] Basheer, Y. et al. Analyzing the Prospect of Hybrid Energy in the Cement Industry of Pakistan, Using HOMER Pro. *Sustainability* **14**, 12440 (2022).

[32] Vargas-Salgado, C., Díaz-Bello, D., Alfonso-Solar, D. & Lara-Vargas, F. Validations of HOMER and SAM tools in predicting energy flows and economic analysis for renewable systems: Comparison to a real-world system result. *Sustain. Energy Technol. Assess.* **69**, 103896 (2024).

[33] Samatar, A. M. et al. Techno-economic and environmental analysis of a fully renewable hybrid energy system for sustainable power infrastructure advancement. *Sci. Rep.* **15**, 12140 (2025). 40204809 10.1038/s41598-025-96401-z PMC11982195

[34] Ngaopitakkul, A. & Yoomak, S. Investigating the feasibility of nano-grid infrastructure integration into street lighting systems based on energy production and economic evaluation. *Sci. Rep.* **14**29833. (2024). 39617753 10.1038/s41598-024-80689-4 PMC11609285

[35] Mumtaz, M. A. et al. Techno-economic and environmental analysis of hybrid energy system for industrial sector of Pakistan. *Sci. Rep.* **14**, 23736 (2024). 39390069 10.1038/s41598-024-74540-z PMC11467415

[36] Molu, R. J. J. et al. A techno-economic perspective on efficient hybrid renewable energy solutions in Douala, Cameroon’s grid-connected systems. *Sci. Rep.* **14**13590. (2024). 38866866 10.1038/s41598-024-64427-4 PMC11637028

[37] Tayyab, Q. et al. Techno-economic configuration of an optimized resident microgrid: A case study for Afghanistan. *Renewable Energy* **224**, 120097 (2024).

[38] Chisale, S. W., Eliya, S. & Taulo, J. Optimization and design of hybrid power system using HOMER pro and integrated CRITIC-PROMETHEE II approaches. *Green Technologies and Sustainability* **1**, 100005 (2023).

[39] Torad, M. M., Elbanna, S. H. A., El-Dabah, M. A. & Zaki Diab, A. A. Optimum sizing of hybrid renewable energy system with biomass backup of Egypt’s Western Desert. *Ain Shams Engineering Journal* **16**, 103402 (2025).

[40] Youssef, A. A., Barakat, S., Tag-Eldin, E. & Samy, M. M. Islanded green energy system optimal analysis using PV, wind, biomass, and battery resources with various economic criteria. *Results in Engineering* **19**, 101321 (2023).

[41] Emamipour, H., Eshghi, M. J. & Khan, A. A. Design of a Hybrid Wind and Micro-Hydro System for Sustainable Water Treatment. *Energies* **18**, 4870 (2025).

[42] Nassar, Y. F. et al. IEEE,. Determination of the Most Accurate Horizontal to Tilted Sky-Diffuse Solar Irradiation Transposition Model for the Capital Cities in MENA Region. In 2022 3rd International Conference on Smart Grid and Renewable Energy (SGRE). 1–6 (2022).

[43] Nassar, Y. F. et al. Sensitivity of global solar irradiance to transposition models: Assessing risks associated with model discrepancies. *e-Prime - Advances in Electrical Engineering, Electronics and Energy* **11**, 100887 (2025).

[44] Ebhota, W. S. & Tabakov, P. Y. Influence of photovoltaic cell technologies and elevated temperature on photovoltaic system performance. *Ain Shams Engineering Journal* **14**, 101984 (2023).

[45] Dubey, S., Sarvaiya, J. N. & Seshadri, B. Temperature Dependent Photovoltaic (PV) Efficiency and Its Effect on PV Production in the World – A Review. *PV Asia Pac. Conf.**2012*** 33**, 311–321 (2013).

[46] Misiani, A. N. & Oni, B. A. A review on challenges in low temperature Lithium-ion cells and future prospects. *Appl. Energy*. **393**, 125987 (2025).

[47] Pamuk, N. Techno-economic feasibility analysis of grid configuration sizing for hybrid renewable energy system in Turkey using different optimization techniques. *Ain Shams Eng. J.* **15**, 102474 (2024).

[48] Khalil, L. et al. Optimization and designing of hybrid power system using HOMER pro. *4th. **Int Conf. Mater. Sci. Nanotechnol*. *MSNANO20.*** 47**, S110–S115 (2021).

[49] Jahangiri, M. et al. Techno–Econo–Enviro Energy Analysis, Ranking and Optimization of Various Building-Integrated Photovoltaic (BIPV) Types in Different Climatic Regions of Iran. *Energies* **16**, 546 (2023).

[50] Elnaggar, M. et al. Leveraging Wind Energy for Electricity and Hydrogen Production: A Sustainable Solution to Power Shortages and Eco-Friendly Alternative Fuel. *Adv. Energy Sustain. Res.* **7**, e202500049 (2026).

[51] Charabi, Y. & Abdul-Wahab, S. Wind turbine performance analysis for energy cost minimization. *Renew. Wind Water Sol*. **7**, 5 (2020).

[52] Ahmad, S., Agrira, A. & Nassar, Y. The Impact of Loss of Power Supply Probability on Design and Performance of Wind/Pumped Hydropower Energy Storage Hybrid System. *Wadi Alshatti Univ. J. Pure Appl. Sci.* **3** (2), 52–62 (2025).

[53] Sobhy, A. & Lei, D. Model-assisted active disturbance rejection controller for maximum efficiency schemes of DFIG‐based wind turbines. *Int Trans. Electr. Energy Syst* **31**, e13107 (2021).

[54] Gopinath, A., Kalyankumar, B. & Quri, K. Techno-Economic Feasibility Analysis of Solar PV- Wind Grid-connected Hybrid Energy systems for Electrification in Sultanate of Oman. *IOP Conf. Ser. Earth Environ. Sci.* **1055**, 012004 (2022).

[55] Imbayah, I. et al. Modeling A 600 MW Floating Photovoltaic System in Al-Khums city, Libya: Performance Analysis and Implementation Using PVSyst. *Wadi Alshatti Univ. J. Pure Appl. Sci.* **4** (1), 223–237 (2026).

[56] Maliat, A., Kotian, S. & Ghahremanlou, D. Assessment of a Hybrid Renewable Energy System Incorporating Wind, Solar, and Storage Technologies in Makkovik, Newfoundland and Labrador. *J. Sustain. Energy*. **3**, 87–104 (2024).

[57] Livinti, P. A. Comparative Study of Storage Batteries for Electrical Energy Produced by Photovoltaic Panels. *Appl. Sci.* **15**, 8549 (2025).

[58] Coccato, S., Barhmi, K., Lampropoulos, I. & Golroodbari, S. Sark, W. A Review of Battery Energy Storage Optimization in the Built Environment. *Batteries* **11**, 179 (2025). van.

[59] Anuphappharadorn, S., Sukchai, S., Sirisamphanwong, C. & Ketjoy, N. Comparison the Economic Analysis of the Battery between Lithium-ion and Lead-acid in PV Stand-alone Application. *Energy Procedia*. **56**, 352–358 (2014).

[60] Ngoy, K. R. et al. Lithium-ion batteries and the future of sustainable energy: A comprehensive review. *Renew. Sustain. Energy Rev.* **223**, 115971 (2025).

[61] Schleifer, A. H., Cohen, S. M., Cole, W. & Denholm, P. Blair, N. Exploring the Future Energy Value of Long-Duration Energy Storage. *Energies* **18**, 1751 (2025).

[62] Abuqila, M., Nassar, Y. & Nyasapoh, M. Estimation of the Storage Capacity of Electric Vehicle Batteries under Real Weather and Drive-mode Conditions: A Case Study. *Wadi Alshatti Univ. J. Pure Appl. Sci.* **3** (1), 58–71 (2025).

[63] Lu, J., Wang, W., Zhang, Y. & Cheng, S. Multi-Objective Optimal Design of Stand-Alone Hybrid Energy System Using Entropy Weight Method Based on HOMER. *Energies* **10**, 1664 (2017).

[64] Palanichamy, C. & Naveen, P. Micro grid for All India Institute of Medical Sciences, Madurai. *Clean Energy* **5**, 254–272 (2021).

[65] Zhou, D., Wang, Z., Xi, K., Zuo, C. & Jia, Y. Optimization Configuration Analysis of Wind-Solar-Storage System Based on HOMER. *Energy Engineering* **122**, 2119–2153 (2025).

[66] Nassar, Y. F. et al. Estimation of CO2 emission within Libya’s electricity generation sector. *Next Research* **2**(3), 100567 (2025).

[67] Eveloy, V. & Gebreegziabher, T. Excess electricity and power-to-gas storage potential in the future renewable-based power generation sector in the United Arab Emirates. *Energy* **166**, 426–450 (2019).

[68] Vaziri Rad, M. A., Kasaeian, A., Niu, X., Zhang, K. & Mahian, O. Excess electricity problem in off-grid hybrid renewable energy systems: A comprehensive review from challenges to prevalent solutions. *Renewable Energy* **212**, 538–560 (2023).

[69] Vaziri Rad, M. A., Kasaeian, A., Mahian, O. & Toopshekan, A. Technical and economic evaluation of excess electricity level management beyond the optimum storage capacity for off-grid renewable systems. *Journal of Energy Storage* **87**, 111385 (2024).

[70] El-Ahmar, M., Ahmed, A. H. & Hemeida, A. *Evaluation Factors Affecting Wind Turbine Output Power. 2017 Nineteenth International Middle East Power Systems Conference (MEPCON),* 1471–1476 (2017).

[71] Lopez-Villalobos, C. A., Martínez-Alvarado, O., Rodriguez-Hernandez, O. & Romero-Centeno, R. Analysis of the influence of the wind speed profile on wind power production. *Energy Rep.* **8**, 8079–8092 (2022).

[72] I Faraz, M. Strategic analysis of wind energy potential and optimal turbine selection in Al-Jouf, Saudi Arabia. *Heliyon* **10**, e39188 (2024). 39502237 10.1016/j.heliyon.2024.e39188 PMC11535323

[73] Said, T. R., Kichonge, B. & Kivevele, T. Optimal design and analysis of a grid-connected hybrid renewable energy system using HOMER Pro: A case study of Tumbatu Island, Zanzibar. *Energy Sci. Eng. ***12**, 2137–2163 (2024).

